# Di-μ_3_-acetato-bis­{μ-6,6′-dieth­oxy-2,2′-[propane-1,2-diylbis(nitrilo­methanylyl­idene)]diphenolato}dicadmiumdisodium ethanol 0.67-solvate

**DOI:** 10.1107/S1600536812047356

**Published:** 2012-11-28

**Authors:** Seik Weng Ng

**Affiliations:** a Department of Chemistry, University of Malaya, 50603 Kuala Lumpur, Malaysia; b Chemistry Department, King Abdulaziz University, PO Box 80203 Jeddah, Saudi Arabia

## Abstract

In the crystal of the title compound, [Cd_2_Na_2_(C_2_H_3_O_2_)_2_(C_21_H_24_N_2_O_4_)_2_]·0.67C_2_H_6_O, the doubly deprotonated Schiff base ligand *N*,*N*′,*O*,*O*′-chelates to the Cd^II^ cation, which is also *O*,*O*′-chelated by the acetate ion. Two Cd–Schiff base units are connected to two Na^+^ atoms to form the tetra­nuclear complex, in which the Cd^2+^ and Na^+^ cations show distorted octa­hedral coordinations. The asymmetric unit consists of half a tetra­nuclear mol­ecule (lying on an inversion center) and a full tetra­nuclear mol­ecule (lying on a general position) along with a lattice ethanol mol­ecule, which links to the coordinating acetate ion *via* an O—H⋯O hydrogen bond. In the crystal, the propyl and ethyl groups of the complex mol­ecule are disordered over two positions in a 1:1 ratio; the ethyl group of the lattice ethanol mol­ecule is also equally disordered over two positions.

## Related literature

For the synthesis and structure of the Schiff base ligand, see: Fun *et al.* (2009 ▶); Jia (2009 ▶).
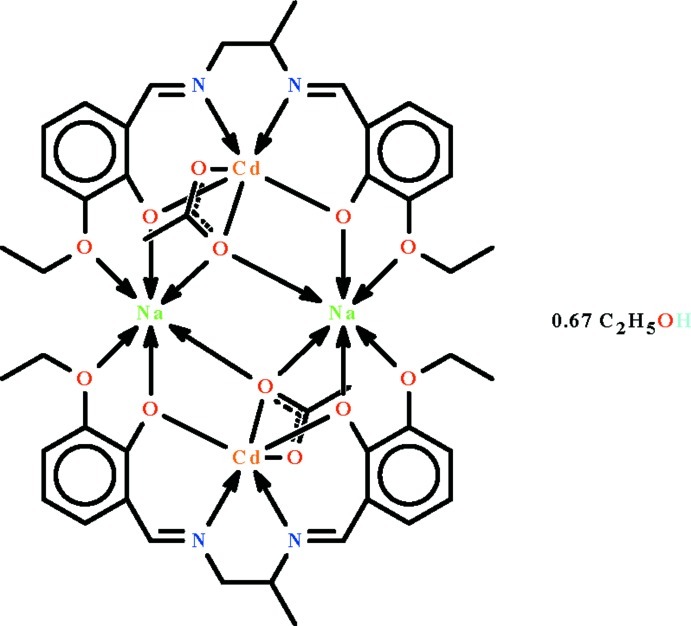


## Experimental

### 

#### Crystal data

[Cd_2_Na_2_(C_2_H_3_O_2_)_2_(C_21_H_24_N_2_O_4_)_2_]·0.67C_2_H_6_O
*M* *_r_* = 1156.42
Triclinic, 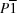

*a* = 13.3568 (8) Å
*b* = 15.0271 (9) Å
*c* = 20.7493 (13) Å
α = 71.359 (2)°
β = 71.551 (2)°
γ = 89.253 (2)°
*V* = 3725.4 (4) Å^3^
*Z* = 3
Mo *K*α radiation
μ = 0.94 mm^−1^
*T* = 100 K
0.15 × 0.10 × 0.10 mm

#### Data collection

Bruker SMART APEX diffractometer
Absorption correction: multi-scan (*SADABS*; Sheldrick, 1996 ▶) *T*_min_ = 0.872, *T*_max_ = 0.912
35751 measured reflections
17030 independent reflections
14709 reflections with *I* > 2σ(*I*)
*R*_int_ = 0.017

#### Refinement

*R*[*F* ^2^ > 2σ(*F* ^2^)] = 0.054
*wR*(*F* ^2^) = 0.147
*S* = 1.05
17030 reflections
897 parameters
176 restraints
H-atom parameters constrained
Δρ_max_ = 2.21 e Å^−3^
Δρ_min_ = −1.50 e Å^−3^

### 

Data collection: *APEX2* (Bruker, 2009 ▶); cell refinement: *SAINT* (Bruker, 2009 ▶); data reduction: *SAINT*; program(s) used to solve structure: *SHELXS97* (Sheldrick, 2008 ▶); program(s) used to refine structure: *SHELXL97* (Sheldrick, 2008 ▶); molecular graphics: *X-SEED* (Barbour, 2001 ▶); software used to prepare material for publication: *publCIF* (Westrip, 2010 ▶).

## Supplementary Material

Crystal structure: contains datablock(s) global, I. DOI: 10.1107/S1600536812047356/xu5642sup1.cif

Structure factors: contains datablock(s) I. DOI: 10.1107/S1600536812047356/xu5642Isup2.hkl

Additional supplementary materials:  crystallographic information; 3D view; checkCIF report

## Figures and Tables

**Table 1:** Hydrogen-bond geometry (Å, °)

*D*—H⋯*A*	*D*—H	H⋯*A*	*D*⋯*A*	*D*—H⋯*A*
O19—H19⋯O17	0.84	1.99	2.730 (7)	146

**Fig. 1.**
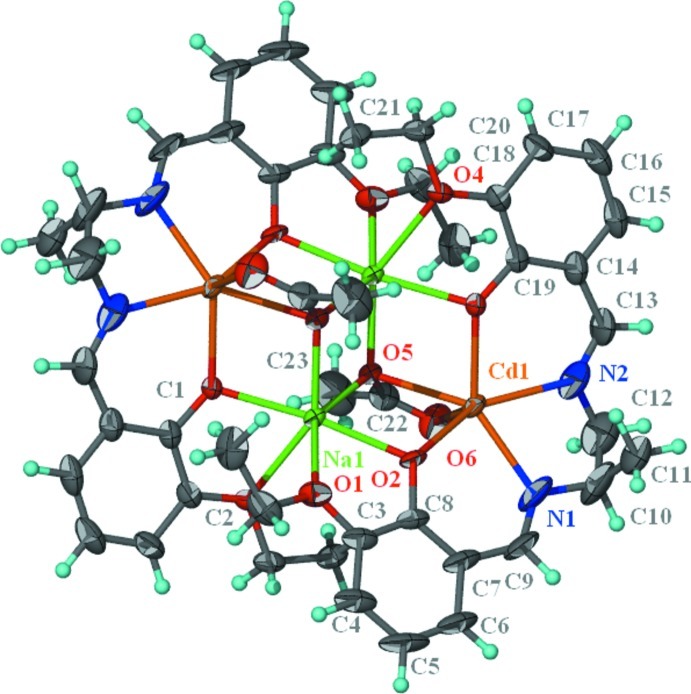
Thermal ellipsoid plot (Barbour, 2001) of [CdNa(CH3CO2)(C21H24N2O4)]2.0.67C2H5OH molecule that lies on a center-of-inversiongeneral position at the 70% probability level; hydrogen atoms are drawn as spheres of arbitrary radius. The disorder component and solvent molecule are not shown.

**Fig. 2.**
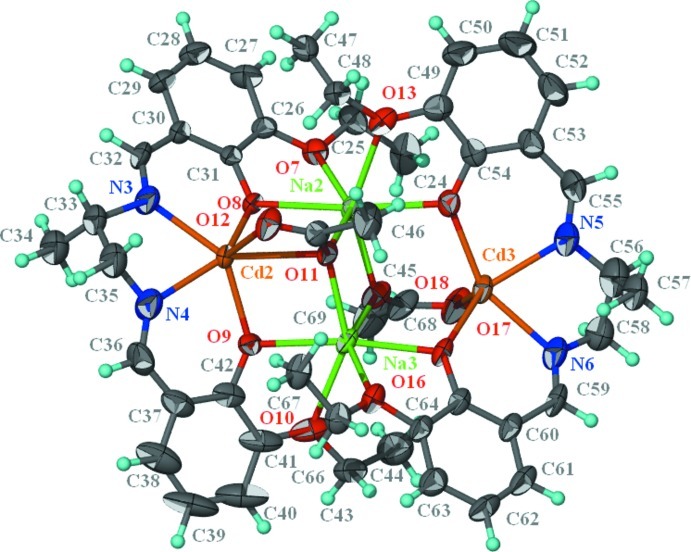
Thermal ellipsoid plot (Barbour, 2001) of [CdNa(CH3CO2)(C21H24N2O4)]2.0.67C2H5OH molecule that lies on a general position at the 70% probability level; hydrogen atoms are drawn as spheres of arbitrary radius. The disorder component and the solvent molecule are not shown.

## supplementary crystallographic information

### Comment

Schiff bases derived from the condensation of salicylaldehyde-type of carbonyl compounds with ethylidenediamine-type of amines commonly *N*,*N*',*O*,*O*'-chelate to metal atoms. The crystal structure of the parent Schiff base in the two polymorphic forms (Fun *et al.*, 2009; Jia, 2009) shows a splayed out conformation so that the deprotonated ligand has to wrap around the larger cadmium atom.

In the present study, the attempt to synthesize the cadmium derivative of the Schiff base required the use of sodium acetate; this reagent was, however, incorporated into the crystal structure along with an ethanol of crystallization (Scheme I). In tetranuclear Na_2_[Cd(CH_3_CO_2_)(C_21_H_24_N_2_O_4_)]_2_^.^0.67C_2_H_5_OH, the doubly-deprotonated Schiff-base ligand *N*,*N*',*O*,*O*'-chelates to cadmium, which is also *O*,*O*'-chelated by the acetate ion. Two Cd—(C_21_H_24_N_2_O_4_) units are connected to two Na atoms; the Cd and Na atoms show octahedral coordination. The asymmetric unit consists of half a tetranculear Na_2_[Cd(CH_3_CO_2_)(C_21_H_24_N_2_O_4_)]_2_ (lying on an inversion center) (Fig. 1) and a full tetranuclear molecule (lying on a general position) (Fig. 2) along with a solvent molecule. This large structure is disordered in several parts.

### Experimental

The Schiff base was synthesized according to the literatue proceduce (Fun *et al.*, 2009; Jia 2009). Cadmium dichloride (1 mmol), sodium acetate (2 mmol) and the Schiff base (1 mmol) were dissolved in 25 mol e thanol. Light yellow crystals were isolated from the filtered solution after several days.

### Refinement

Carbon-bound H-atoms were placed in calculated positions (C—H 0.95 to 0.99 Å) and were included in the refinement in the riding model approximation, with *U*(H) set to 1.2 to 1.5*U*(C).

The hydroxy H-atom was placed in a calculated position (O–H 0.84 Å) and its temperature factor tied by a factor of 1.5 times.

The asymmetric unit consists of a CdNa(CH_3_CO_2_)(C_21_H_24_N_2_O_4_)molecule that is near a center-of-inversion (that results in the formation of a centrosymmetric dimer), a dinculear [CdNa(CH_3_CO_2_)(C_21_H_24_N_2_O_4_)]_2_ molecule (that lies on a general position) and an ethanol of crystallization. In the both, the carbon segment of the N–CH_2_–CH(CH_3_)–N fragment is disordered over two positions. However, in the second dimer, one ethoxy group of each C_21_H_24_N_2_O_4_ dianion is disordered over two positions, one in respect of the ethoxy group and the other in respect of the ethyl fragment only. The solvent molecule is also disordered in the ethyl fragment. As the occupancy refined to nearly 1:1, the occupancy was then fixed as exactly 0.5 for all components.

To treat the disorder, carbon-carbon distances were restrained to 1.50±0.01 Å, carbon–nitrogen distances to 1.45±0.01 Å and carbon-oxygen distances to 1.45±0.01 Å. The temperature factors of the primed atoms were set to those of the unprimed ones; the anisotropic temperature factors were also tightly restrained to be nearly isotropic.

The nitrogen atoms that are involved in coordination were also tightly restrained in the anisotropic temperature factors.

Omitted from the refinement were the reflections seriously affected by the beam stop: (1 0 0), (0 1 0), (0 0 1), (1 1 0), (0 1 1) and (1 0 1).

The final difference Fourier map had a peak at 0.80 Å from Cd3 and a hole at 0.68 Å from the same atom.

### Crystal data

**Table:**

[Cd_2_Na_2_(C_2_H_3_O_2_)_2_(C_21_H_24_N_2_O_4_)_2_]·0.67C_2_H_6_O	*Z* = 3
*M**_r_* = 1156.42	*F*(000) = 1768
Triclinic, *P*1	*D*_x_ = 1.546 Mg m^−^^3^
Hall symbol: -P 1	Mo *K*α radiation, λ = 0.71073 Å
*a* = 13.3568 (8) Å	Cell parameters from 9846 reflections
*b* = 15.0271 (9) Å	θ = 2.3–28.3°
*c* = 20.7493 (13) Å	µ = 0.94 mm^−^^1^
α = 71.359 (2)°	*T* = 100 K
β = 71.551 (2)°	Prism, light yellow
γ = 89.253 (2)°	0.15 × 0.10 × 0.10 mm
*V* = 3725.4 (4) Å^3^	

### Data collection

**Table:**

Bruker SMART APEX diffractometer	17030 independent reflections
Radiation source: fine-focus sealed tube	14709 reflections with *I* > 2σ(*I*)
Graphite monochromator	*R*_int_ = 0.017
ω scans	θ_max_ = 27.5°, θ_min_ = 2.1°
Absorption correction: multi-scan (*SADABS*; Sheldrick, 1996)	*h* = −17→17
*T*_min_ = 0.872, *T*_max_ = 0.912	*k* = −19→19
35751 measured reflections	*l* = −26→26

### Refinement

**Table:**

Refinement on *F*^2^	Primary atom site location: structure-invariant direct methods
Least-squares matrix: full	Secondary atom site location: difference Fourier map
*R*[*F*^2^ > 2σ(*F*^2^)] = 0.054	Hydrogen site location: inferred from neighbouring sites
*wR*(*F*^2^) = 0.147	H-atom parameters constrained
*S* = 1.05	*w* = 1/[σ^2^(*F*_o_^2^) + (0.0689*P*)^2^ + 10.728*P*] where *P* = (*F*_o_^2^ + 2*F*_c_^2^)/3
17030 reflections	(Δ/σ)_max_ = 0.001
897 parameters	Δρ_max_ = 2.21 e Å^−^^3^
176 restraints	Δρ_min_ = −1.50 e Å^−^^3^

### Fractional atomic coordinates and isotropic or equivalent isotropic displacement parameters (Å^2^)

**Table:**

	*x*	*y*	*z*	*U*_iso_*/*U*_eq_	Occ. (<1)
Cd1	1.03035 (3)	0.38345 (2)	0.396852 (17)	0.03411 (9)	
Cd2	−0.06583 (3)	0.98869 (2)	0.746595 (16)	0.03469 (9)	
Cd3	0.18049 (3)	0.78325 (2)	0.958660 (19)	0.04274 (10)	
Na1	0.86760 (12)	0.48273 (12)	0.52155 (9)	0.0318 (3)	
Na2	0.17651 (15)	0.93330 (13)	0.78200 (10)	0.0415 (4)	
Na3	−0.04807 (17)	0.81139 (12)	0.90295 (9)	0.0429 (4)	
O1	0.7212 (3)	0.3595 (2)	0.59619 (18)	0.0459 (8)	
O2	0.8772 (2)	0.3497 (2)	0.48667 (17)	0.0341 (6)	
O3	1.1635 (2)	0.3880 (2)	0.43970 (16)	0.0338 (6)	
O4	1.2798 (2)	0.4262 (2)	0.50874 (17)	0.0355 (6)	
O5	1.0114 (2)	0.5363 (2)	0.41665 (17)	0.0344 (6)	
O6	1.0231 (4)	0.5266 (3)	0.3113 (2)	0.0601 (10)	
O7	0.2998 (3)	0.9376 (2)	0.6632 (2)	0.0473 (8)	
O8	0.1020 (2)	0.9709 (2)	0.68944 (15)	0.0363 (7)	
O9	−0.1400 (3)	0.8480 (2)	0.82239 (17)	0.0410 (7)	
O10	−0.1663 (4)	0.6828 (3)	0.9197 (2)	0.0723 (13)	
O11	0.0123 (3)	0.9683 (2)	0.84160 (16)	0.0380 (7)	
O12	−0.0668 (4)	1.0948 (2)	0.8062 (2)	0.0562 (10)	
O13	0.2671 (9)	1.0830 (8)	0.7738 (7)	0.045 (2)	0.50
O14	0.2510 (3)	0.9186 (2)	0.8728 (2)	0.0469 (8)	
O15	0.0078 (3)	0.7742 (2)	1.00104 (17)	0.0440 (8)	
O16	−0.1847 (3)	0.8139 (3)	1.01538 (18)	0.0508 (9)	
O17	0.2258 (4)	0.6807 (3)	0.8973 (2)	0.0655 (12)	
O18	0.1229 (3)	0.7726 (2)	0.8461 (2)	0.0536 (9)	
O19	0.3700 (3)	0.5538 (3)	0.9191 (2)	0.0647 (11)	
H19	0.3384	0.6020	0.9216	0.097*	
N1	0.9465 (5)	0.2741 (5)	0.3691 (3)	0.0831 (18)	
N2	1.1589 (6)	0.3265 (5)	0.3226 (4)	0.091 (2)	
N3	−0.0542 (4)	1.0849 (4)	0.6337 (3)	0.0657 (14)	
N4	−0.2346 (4)	0.9964 (5)	0.7381 (3)	0.0795 (17)	
N5	0.3211 (5)	0.8133 (4)	0.9938 (3)	0.0805 (17)	
N6	0.1538 (4)	0.6793 (4)	1.0724 (3)	0.0692 (14)	
C1	0.6937 (6)	0.3894 (5)	0.7061 (3)	0.0687 (18)	
H1A	0.6406	0.3869	0.7520	0.103*	
H1B	0.7250	0.4546	0.6790	0.103*	
H1C	0.7494	0.3492	0.7155	0.103*	
C2	0.6421 (4)	0.3551 (4)	0.6633 (3)	0.0609 (15)	
H2A	0.6100	0.2893	0.6904	0.073*	
H2B	0.5852	0.3949	0.6540	0.073*	
C3	0.6979 (2)	0.3133 (2)	0.55485 (17)	0.0455 (12)	
C4	0.5973 (2)	0.2725 (3)	0.56842 (19)	0.0617 (16)	
H4	0.5394	0.2776	0.6075	0.074*	
C5	0.5815 (2)	0.2242 (3)	0.5249 (2)	0.082 (3)	
H5	0.5127	0.1963	0.5341	0.098*	
C6	0.6662 (3)	0.2168 (2)	0.4677 (2)	0.0669 (19)	
H6	0.6554	0.1838	0.4380	0.080*	
C7	0.7668 (3)	0.2576 (2)	0.45416 (17)	0.0549 (15)	
C8	0.7827 (2)	0.3059 (2)	0.49772 (17)	0.0394 (10)	
C9	0.8499 (6)	0.2395 (5)	0.3953 (4)	0.0722 (19)	
H9	0.8297	0.1978	0.3745	0.087*	
C10	1.0236 (11)	0.2489 (11)	0.3104 (7)	0.097 (4)	0.50
H10	0.9882	0.2330	0.2791	0.116*	0.50
C11	1.0697 (13)	0.1638 (9)	0.3495 (7)	0.083 (3)	0.50
H11A	1.0128	0.1129	0.3795	0.125*	0.50
H11B	1.1032	0.1813	0.3801	0.125*	0.50
H11C	1.1227	0.1423	0.3142	0.125*	0.50
C12	1.1181 (16)	0.3192 (11)	0.2667 (7)	0.099 (4)	0.50
H12A	1.1705	0.2960	0.2314	0.118*	0.50
H12B	1.0976	0.3805	0.2410	0.118*	0.50
C13	1.2576 (6)	0.3187 (6)	0.3159 (4)	0.078 (2)	
H13	1.2975	0.2924	0.2809	0.094*	
C14	1.3118 (2)	0.3462 (2)	0.35648 (16)	0.0472 (11)	
C15	1.4201 (3)	0.3379 (3)	0.33336 (16)	0.0584 (15)	
H15	1.4507	0.3174	0.2932	0.070*	
C16	1.48359 (18)	0.3595 (3)	0.3690 (2)	0.0641 (17)	
H16	1.5576	0.3538	0.3533	0.077*	
C17	1.4388 (2)	0.3895 (3)	0.42785 (19)	0.0514 (13)	
H17	1.4822	0.4043	0.4522	0.062*	
C18	1.3305 (2)	0.3978 (2)	0.45097 (14)	0.0375 (9)	
C19	1.26703 (17)	0.3762 (2)	0.41529 (16)	0.0359 (9)	
C20	1.3438 (4)	0.4527 (4)	0.5448 (3)	0.0440 (11)	
H20A	1.3942	0.5082	0.5114	0.053*	
H20B	1.3849	0.4001	0.5614	0.053*	
C21	1.2722 (4)	0.4760 (3)	0.6078 (3)	0.0433 (11)	
H21A	1.3148	0.4938	0.6334	0.065*	
H21B	1.2224	0.4208	0.6405	0.065*	
H21C	1.2326	0.5288	0.5908	0.065*	
C22	1.0135 (4)	0.5736 (3)	0.3523 (3)	0.0459 (11)	
C23	1.0023 (7)	0.6783 (4)	0.3259 (5)	0.091 (3)	
H23A	0.9476	0.6947	0.3635	0.136*	
H23B	0.9819	0.6939	0.2825	0.136*	
H23C	1.0701	0.7139	0.3147	0.136*	
C24	0.4195 (6)	0.8563 (6)	0.7188 (4)	0.076 (2)	
H24A	0.4944	0.8476	0.7127	0.115*	
H24B	0.3812	0.7961	0.7277	0.115*	
H24C	0.3888	0.8786	0.7597	0.115*	
C25	0.4107 (4)	0.9272 (4)	0.6522 (3)	0.0546 (13)	
H25A	0.4492	0.9883	0.6426	0.065*	
H25B	0.4420	0.9055	0.6103	0.065*	
C26	0.2737 (2)	1.0011 (2)	0.60811 (16)	0.0414 (10)	
C27	0.34796 (19)	1.0500 (3)	0.54160 (18)	0.0565 (14)	
H27	0.4203	1.0378	0.5317	0.068*	
C28	0.3164 (2)	1.1166 (3)	0.48959 (14)	0.0633 (16)	
H28	0.3671	1.1501	0.4441	0.076*	
C29	0.2106 (3)	1.1344 (2)	0.50408 (15)	0.0577 (14)	
H29	0.1890	1.1800	0.4685	0.069*	
C30	0.13632 (19)	1.0855 (2)	0.57058 (16)	0.0451 (11)	
C31	0.1679 (2)	1.0189 (2)	0.62260 (12)	0.0371 (9)	
C32	0.0296 (5)	1.1133 (5)	0.5777 (3)	0.0631 (17)	
H32	0.0207	1.1578	0.5360	0.076*	
C33	−0.1533 (7)	1.1080 (10)	0.6185 (6)	0.065 (3)	0.50
H33	−0.1453	1.1760	0.5882	0.078*	0.50
C34	−0.1884 (13)	1.0513 (12)	0.5831 (7)	0.083 (3)	0.50
H34A	−0.1356	1.0610	0.5356	0.124*	0.50
H34B	−0.2565	1.0700	0.5774	0.124*	0.50
H34C	−0.1968	0.9844	0.6125	0.124*	0.50
C35	−0.2351 (11)	1.0942 (8)	0.6901 (5)	0.077 (3)	0.50
H35A	−0.3059	1.1035	0.6848	0.093*	0.50
H35B	−0.2187	1.1406	0.7111	0.093*	0.50
C36	−0.3148 (5)	0.9325 (6)	0.7721 (4)	0.078 (2)	
H36	−0.3771	0.9471	0.7597	0.094*	
C37	−0.3215 (3)	0.8418 (3)	0.8264 (2)	0.0639 (17)	
C38	−0.4208 (3)	0.7908 (4)	0.8566 (3)	0.099 (3)	
H38	−0.4782	0.8164	0.8414	0.118*	
C39	−0.4360 (3)	0.7023 (4)	0.9089 (3)	0.121 (4)	
H39	−0.5039	0.6674	0.9295	0.145*	
C40	−0.3520 (4)	0.6649 (3)	0.9311 (3)	0.104 (3)	
H40	−0.3624	0.6044	0.9668	0.125*	
C41	−0.2528 (3)	0.7159 (3)	0.9009 (2)	0.073 (2)	
C42	−0.2375 (2)	0.8044 (3)	0.8486 (2)	0.0511 (13)	
C43	−0.1590 (10)	0.5889 (8)	0.9701 (8)	0.062 (3)	0.50
H43A	−0.1948	0.5408	0.9594	0.074*	0.50
H43B	−0.2005	0.5870	1.0195	0.074*	0.50
C44	−0.0521 (12)	0.5591 (14)	0.9709 (11)	0.086 (3)	0.50
H44A	−0.0602	0.4957	1.0060	0.129*	0.50
H44B	−0.0164	0.6035	0.9843	0.129*	0.50
H44C	−0.0097	0.5586	0.9229	0.129*	0.50
C45	−0.0190 (5)	1.0469 (3)	0.8456 (3)	0.0468 (12)	
C46	0.0037 (7)	1.0817 (5)	0.9010 (4)	0.075 (2)	
H46A	0.0748	1.0672	0.9027	0.112*	
H46B	−0.0490	1.0503	0.9485	0.112*	
H46C	0.0000	1.1501	0.8877	0.112*	
C47	0.2941 (9)	1.2067 (8)	0.6528 (5)	0.0560 (19)	0.50
H47A	0.2645	1.2637	0.6304	0.084*	0.50
H47B	0.2903	1.1596	0.6299	0.084*	0.50
H47C	0.3683	1.2224	0.6466	0.084*	0.50
C48	0.2323 (8)	1.1678 (6)	0.7310 (5)	0.0451 (16)	0.50
H48A	0.2336	1.2178	0.7523	0.054*	0.50
H48B	0.1576	1.1533	0.7359	0.054*	0.50
C49	0.3490 (3)	1.0626 (3)	0.80267 (18)	0.0534 (13)	
C50	0.4344 (4)	1.1281 (3)	0.7844 (2)	0.088 (3)	
H50	0.4448	1.1854	0.7453	0.105*	
C51	0.5045 (3)	1.1098 (4)	0.8232 (2)	0.116 (4)	
H51	0.5629	1.1546	0.8107	0.139*	
C52	0.4893 (3)	1.0260 (4)	0.8803 (2)	0.087 (3)	
H52	0.5372	1.0135	0.9068	0.104*	
C53	0.4038 (3)	0.9605 (3)	0.89861 (19)	0.0570 (14)	
C54	0.3337 (2)	0.9787 (2)	0.85979 (19)	0.0443 (11)	
C55	0.3941 (5)	0.8812 (6)	0.9618 (4)	0.079 (2)	
H55	0.4488	0.8790	0.9822	0.095*	
C56	0.3205 (16)	0.7377 (10)	1.0596 (7)	0.100 (3)	0.50
H56	0.3944	0.7267	1.0599	0.120*	0.50
C57	0.2597 (15)	0.7712 (12)	1.1210 (10)	0.083 (3)	0.50
H57A	0.2962	0.8301	1.1167	0.124*	0.50
H57B	0.2554	0.7231	1.1670	0.124*	0.50
H57C	0.1879	0.7822	1.1193	0.124*	0.50
C58	0.2585 (9)	0.6476 (11)	1.0745 (9)	0.070 (4)	0.50
H58A	0.2903	0.6185	1.0370	0.084*	0.50
H58B	0.2531	0.6020	1.1223	0.084*	0.50
C59	0.0675 (5)	0.6449 (4)	1.1228 (3)	0.0581 (15)	
H59	0.0731	0.6042	1.1669	0.070*	
C60	−0.0402 (2)	0.6607 (2)	1.12096 (17)	0.0475 (12)	
C61	−0.1212 (3)	0.6108 (2)	1.18367 (14)	0.0569 (15)	
H61	−0.1048	0.5655	1.2220	0.068*	
C62	−0.2261 (3)	0.6271 (2)	1.19033 (14)	0.0595 (16)	
H62	−0.2814	0.5929	1.2332	0.071*	
C63	−0.2500 (2)	0.6933 (3)	1.13429 (18)	0.0582 (14)	
H63	−0.3217	0.7044	1.1388	0.070*	
C64	−0.1691 (3)	0.7432 (2)	1.07158 (14)	0.0455 (11)	
C65	−0.0642 (2)	0.7270 (2)	1.06491 (13)	0.0425 (11)	
C66	−0.2863 (5)	0.8479 (4)	1.0249 (3)	0.0549 (13)	
H66A	−0.3135	0.8578	1.0721	0.066*	
H66B	−0.3368	0.8014	1.0236	0.066*	
C67	−0.2755 (5)	0.9387 (4)	0.9656 (3)	0.0586 (14)	
H67A	−0.3451	0.9630	0.9708	0.088*	
H67B	−0.2481	0.9283	0.9191	0.088*	
H67C	−0.2264	0.9846	0.9678	0.088*	
C68	0.1780 (6)	0.7045 (4)	0.8514 (3)	0.0600 (16)	
C69	0.1894 (8)	0.6469 (6)	0.8019 (4)	0.095 (3)	
H69A	0.1720	0.6839	0.7592	0.142*	
H69B	0.1410	0.5890	0.8274	0.142*	
H69C	0.2626	0.6307	0.7872	0.142*	
C70	0.4124 (13)	0.5377 (12)	0.8513 (6)	0.080 (3)	0.50
H70A	0.4426	0.5977	0.8115	0.097*	0.50
H70B	0.3570	0.5072	0.8409	0.097*	0.50
C71	0.4968 (16)	0.4739 (15)	0.8615 (13)	0.104 (5)	0.50
H71A	0.5314	0.4601	0.8169	0.156*	0.50
H71B	0.4650	0.4149	0.9008	0.156*	0.50
H71C	0.5494	0.5047	0.8733	0.156*	0.50
O13'	0.2838 (10)	1.0734 (8)	0.7613 (7)	0.045 (2)	0.50
C10'	1.0163 (13)	0.2196 (9)	0.3295 (8)	0.097 (4)	0.50
H10B	0.9820	0.2003	0.2995	0.116*	0.50
H10C	1.0312	0.1620	0.3635	0.116*	0.50
C11'	1.1161 (12)	0.2803 (10)	0.2833 (8)	0.083 (3)	0.50
H11'	1.1699	0.2460	0.2571	0.100*	0.50
C12'	1.0774 (15)	0.3605 (10)	0.2315 (8)	0.099 (4)	0.50
H12C	1.1383	0.4046	0.1965	0.148*	0.50
H12D	1.0275	0.3940	0.2591	0.148*	0.50
H12E	1.0420	0.3338	0.2061	0.148*	0.50
C33'	−0.1538 (7)	1.1340 (8)	0.6426 (7)	0.065 (3)	0.50
H33B	−0.1529	1.1803	0.5961	0.078*	0.50
H33C	−0.1621	1.1672	0.6780	0.078*	0.50
C34'	−0.2418 (11)	1.0565 (9)	0.6694 (5)	0.083 (3)	0.50
H34'	−0.3109	1.0848	0.6787	0.099*	0.50
C35'	−0.2249 (12)	1.0239 (11)	0.6053 (8)	0.077 (3)	0.50
H35C	−0.2793	0.9726	0.6175	0.116*	0.50
H35D	−0.1546	1.0013	0.5926	0.116*	0.50
H35E	−0.2300	1.0767	0.5644	0.116*	0.50
C56'	0.3217 (17)	0.7587 (12)	1.0664 (6)	0.100 (3)	0.50
H56'	0.3954	0.7427	1.0639	0.120*	0.50
C43'	−0.2037 (10)	0.5897 (7)	0.9698 (8)	0.062 (3)	0.50
H43C	−0.2430	0.5527	0.9521	0.074*	0.50
H43D	−0.2479	0.5902	1.0180	0.074*	0.50
C44'	−0.1002 (12)	0.5566 (14)	0.9692 (12)	0.086 (3)	0.50
H44D	−0.1099	0.4917	1.0025	0.129*	0.50
H44E	−0.0625	0.5974	0.9843	0.129*	0.50
H44F	−0.0588	0.5583	0.9204	0.129*	0.50
C47'	0.1999 (8)	1.1895 (8)	0.6973 (7)	0.0560 (19)	0.50
H47D	0.2083	1.2521	0.6609	0.084*	0.50
H47E	0.1424	1.1872	0.7413	0.084*	0.50
H47F	0.1826	1.1414	0.6789	0.084*	0.50
C48'	0.3016 (8)	1.1711 (6)	0.7139 (5)	0.0451 (16)	0.50
H48C	0.3621	1.1798	0.6692	0.054*	0.50
H48D	0.3159	1.2140	0.7382	0.054*	0.50
C57'	0.2763 (18)	0.7969 (14)	1.1273 (12)	0.100 (3)	0.50
H57D	0.3152	0.8577	1.1161	0.150*	0.50
H57E	0.2828	0.7523	1.1717	0.150*	0.50
H57F	0.2013	0.8054	1.1337	0.150*	0.50
C58'	0.2512 (11)	0.6699 (11)	1.0909 (11)	0.083 (3)	0.50
H58C	0.2922	0.6246	1.0700	0.099*	0.50
H58D	0.2316	0.6422	1.1439	0.099*	0.50
C70'	0.4573 (10)	0.5599 (12)	0.8546 (7)	0.080 (3)	0.50
H70C	0.4937	0.6247	0.8327	0.097*	0.50
H70D	0.4295	0.5469	0.8193	0.097*	0.50
C71'	0.5348 (16)	0.4910 (15)	0.8714 (13)	0.104 (5)	0.50
H71D	0.5928	0.4963	0.8269	0.156*	0.50
H71E	0.4989	0.4269	0.8926	0.156*	0.50
H71F	0.5634	0.5048	0.9056	0.156*	0.50

### Atomic displacement parameters (Å^2^)

**Table:**

	*U*^11^	*U*^22^	*U*^33^	*U*^12^	*U*^13^	*U*^23^
Cd1	0.04321 (18)	0.03671 (17)	0.03397 (16)	0.00355 (13)	−0.01944 (13)	−0.02036 (13)
Cd2	0.03925 (17)	0.03878 (17)	0.02801 (15)	0.00378 (13)	−0.01704 (13)	−0.00780 (12)
Cd3	0.0615 (2)	0.03331 (17)	0.04287 (19)	0.01652 (15)	−0.03101 (17)	−0.01193 (14)
Na1	0.0275 (8)	0.0355 (8)	0.0398 (9)	0.0010 (6)	−0.0123 (7)	−0.0210 (7)
Na2	0.0511 (11)	0.0358 (9)	0.0397 (9)	0.0089 (8)	−0.0246 (8)	−0.0058 (7)
Na3	0.0635 (12)	0.0334 (9)	0.0298 (9)	0.0043 (8)	−0.0205 (8)	−0.0029 (7)
O1	0.0356 (17)	0.0483 (19)	0.0443 (18)	−0.0107 (14)	−0.0079 (14)	−0.0074 (15)
O2	0.0344 (15)	0.0329 (15)	0.0447 (17)	−0.0042 (12)	−0.0224 (13)	−0.0160 (13)
O3	0.0298 (14)	0.0446 (17)	0.0361 (15)	0.0092 (12)	−0.0116 (12)	−0.0248 (13)
O4	0.0273 (14)	0.0431 (17)	0.0412 (16)	0.0036 (12)	−0.0149 (12)	−0.0173 (14)
O5	0.0306 (15)	0.0360 (15)	0.0432 (17)	0.0010 (12)	−0.0138 (13)	−0.0205 (13)
O6	0.086 (3)	0.059 (2)	0.0393 (19)	0.011 (2)	−0.030 (2)	−0.0132 (18)
O7	0.0414 (18)	0.0452 (19)	0.053 (2)	0.0094 (15)	−0.0158 (16)	−0.0131 (16)
O8	0.0407 (16)	0.0369 (16)	0.0271 (14)	−0.0022 (13)	−0.0124 (12)	−0.0035 (12)
O9	0.0492 (19)	0.0413 (17)	0.0335 (16)	−0.0083 (14)	−0.0129 (14)	−0.0138 (13)
O10	0.089 (3)	0.0356 (19)	0.066 (3)	−0.009 (2)	0.002 (2)	−0.0081 (19)
O11	0.0550 (19)	0.0307 (15)	0.0338 (15)	0.0039 (13)	−0.0225 (14)	−0.0103 (12)
O12	0.091 (3)	0.0356 (18)	0.054 (2)	0.0202 (18)	−0.038 (2)	−0.0166 (16)
O13	0.057 (3)	0.036 (2)	0.039 (4)	0.017 (2)	−0.008 (3)	−0.015 (2)
O14	0.056 (2)	0.0351 (17)	0.057 (2)	0.0094 (15)	−0.0331 (18)	−0.0104 (15)
O15	0.065 (2)	0.0385 (17)	0.0294 (15)	0.0123 (15)	−0.0224 (15)	−0.0064 (13)
O16	0.064 (2)	0.052 (2)	0.0366 (17)	0.0135 (17)	−0.0212 (16)	−0.0095 (15)
O17	0.113 (4)	0.040 (2)	0.051 (2)	0.033 (2)	−0.036 (2)	−0.0188 (17)
O18	0.075 (3)	0.0345 (17)	0.0440 (19)	0.0109 (17)	−0.0174 (18)	−0.0063 (15)
O19	0.060 (2)	0.054 (2)	0.063 (3)	0.0015 (19)	−0.002 (2)	−0.014 (2)
N1	0.096 (4)	0.093 (3)	0.086 (3)	−0.014 (3)	−0.027 (3)	−0.066 (3)
N2	0.098 (4)	0.128 (4)	0.087 (3)	0.018 (3)	−0.034 (3)	−0.083 (3)
N3	0.046 (2)	0.074 (3)	0.059 (3)	0.008 (2)	−0.025 (2)	0.009 (2)
N4	0.051 (3)	0.100 (4)	0.066 (3)	0.009 (2)	−0.024 (2)	0.007 (3)
N5	0.068 (3)	0.084 (3)	0.083 (3)	0.012 (3)	−0.049 (3)	0.004 (3)
N6	0.071 (3)	0.068 (3)	0.064 (3)	0.016 (2)	−0.038 (2)	0.001 (2)
C1	0.082 (4)	0.059 (4)	0.041 (3)	0.001 (3)	0.005 (3)	−0.009 (3)
C2	0.043 (3)	0.059 (3)	0.055 (3)	0.001 (2)	0.001 (2)	−0.001 (3)
C3	0.039 (2)	0.034 (2)	0.059 (3)	−0.0055 (19)	−0.027 (2)	0.002 (2)
C4	0.044 (3)	0.047 (3)	0.085 (4)	−0.010 (2)	−0.035 (3)	0.003 (3)
C5	0.069 (4)	0.052 (3)	0.132 (7)	−0.018 (3)	−0.074 (5)	−0.001 (4)
C6	0.075 (4)	0.045 (3)	0.105 (5)	−0.004 (3)	−0.066 (4)	−0.021 (3)
C7	0.069 (4)	0.034 (2)	0.079 (4)	−0.005 (2)	−0.054 (3)	−0.012 (2)
C8	0.044 (2)	0.0239 (19)	0.056 (3)	−0.0022 (17)	−0.035 (2)	−0.0026 (18)
C9	0.091 (5)	0.066 (4)	0.097 (5)	−0.001 (3)	−0.056 (4)	−0.051 (4)
C10	0.125 (6)	0.093 (6)	0.093 (5)	−0.013 (4)	−0.024 (4)	−0.069 (5)
C11	0.096 (5)	0.101 (5)	0.082 (5)	0.017 (4)	−0.034 (4)	−0.066 (4)
C12	0.122 (6)	0.102 (6)	0.088 (6)	0.012 (4)	−0.038 (4)	−0.049 (4)
C13	0.067 (4)	0.120 (6)	0.071 (4)	0.026 (4)	−0.012 (3)	−0.073 (5)
C14	0.048 (3)	0.044 (3)	0.042 (3)	0.005 (2)	−0.001 (2)	−0.018 (2)
C15	0.059 (3)	0.049 (3)	0.050 (3)	0.014 (3)	0.009 (3)	−0.019 (2)
C16	0.036 (3)	0.061 (3)	0.072 (4)	0.012 (2)	0.007 (3)	−0.016 (3)
C17	0.028 (2)	0.051 (3)	0.068 (3)	0.005 (2)	−0.009 (2)	−0.016 (3)
C18	0.032 (2)	0.033 (2)	0.042 (2)	0.0047 (17)	−0.0070 (18)	−0.0095 (18)
C19	0.037 (2)	0.031 (2)	0.034 (2)	0.0047 (17)	−0.0047 (18)	−0.0112 (17)
C20	0.038 (2)	0.046 (3)	0.055 (3)	0.004 (2)	−0.029 (2)	−0.012 (2)
C21	0.052 (3)	0.041 (2)	0.043 (3)	0.000 (2)	−0.028 (2)	−0.010 (2)
C22	0.045 (3)	0.037 (2)	0.051 (3)	0.003 (2)	−0.018 (2)	−0.006 (2)
C23	0.090 (5)	0.040 (3)	0.114 (6)	0.012 (3)	−0.025 (5)	0.004 (4)
C24	0.071 (4)	0.093 (5)	0.082 (5)	0.052 (4)	−0.034 (4)	−0.045 (4)
C25	0.042 (3)	0.058 (3)	0.071 (4)	0.012 (2)	−0.020 (3)	−0.030 (3)
C26	0.048 (3)	0.031 (2)	0.041 (2)	−0.0031 (19)	−0.014 (2)	−0.0087 (19)
C27	0.045 (3)	0.053 (3)	0.058 (3)	−0.004 (2)	−0.005 (2)	−0.014 (3)
C28	0.061 (4)	0.062 (4)	0.044 (3)	−0.014 (3)	−0.003 (3)	−0.002 (3)
C29	0.063 (3)	0.054 (3)	0.040 (3)	−0.012 (3)	−0.016 (2)	0.006 (2)
C30	0.049 (3)	0.049 (3)	0.033 (2)	−0.008 (2)	−0.018 (2)	−0.004 (2)
C31	0.045 (2)	0.031 (2)	0.035 (2)	−0.0076 (18)	−0.0162 (19)	−0.0074 (17)
C32	0.053 (3)	0.076 (4)	0.042 (3)	−0.002 (3)	−0.026 (2)	0.014 (3)
C33	0.058 (3)	0.074 (5)	0.059 (5)	0.005 (3)	−0.033 (3)	−0.003 (3)
C34	0.076 (5)	0.096 (5)	0.074 (5)	0.004 (4)	−0.036 (4)	−0.013 (4)
C35	0.071 (5)	0.091 (5)	0.072 (4)	0.012 (4)	−0.037 (4)	−0.016 (4)
C36	0.035 (3)	0.137 (7)	0.057 (4)	−0.004 (3)	−0.012 (3)	−0.027 (4)
C37	0.046 (3)	0.088 (5)	0.059 (4)	−0.014 (3)	−0.002 (3)	−0.039 (3)
C38	0.047 (4)	0.113 (7)	0.128 (7)	−0.013 (4)	0.000 (4)	−0.058 (6)
C39	0.070 (5)	0.098 (7)	0.160 (10)	−0.046 (5)	0.018 (6)	−0.052 (7)
C40	0.077 (5)	0.065 (5)	0.129 (8)	−0.027 (4)	0.023 (5)	−0.033 (5)
C41	0.072 (4)	0.053 (3)	0.075 (4)	−0.025 (3)	0.015 (3)	−0.033 (3)
C42	0.051 (3)	0.055 (3)	0.046 (3)	−0.009 (2)	0.001 (2)	−0.032 (2)
C43	0.053 (5)	0.050 (3)	0.066 (4)	−0.006 (4)	−0.008 (4)	−0.008 (3)
C44	0.092 (6)	0.072 (4)	0.087 (5)	0.004 (5)	−0.027 (4)	−0.017 (4)
C45	0.068 (3)	0.039 (2)	0.040 (2)	−0.003 (2)	−0.023 (2)	−0.016 (2)
C46	0.118 (6)	0.065 (4)	0.071 (4)	0.011 (4)	−0.052 (4)	−0.042 (3)
C47	0.060 (4)	0.044 (3)	0.066 (4)	−0.001 (3)	−0.033 (3)	−0.009 (3)
C48	0.044 (3)	0.043 (3)	0.044 (3)	0.001 (3)	−0.011 (3)	−0.012 (3)
C49	0.058 (3)	0.057 (3)	0.040 (3)	0.000 (3)	−0.005 (2)	−0.020 (2)
C50	0.098 (6)	0.095 (5)	0.047 (3)	−0.044 (5)	−0.009 (4)	−0.004 (3)
C51	0.093 (6)	0.169 (10)	0.054 (4)	−0.079 (6)	−0.003 (4)	−0.013 (5)
C52	0.043 (3)	0.148 (8)	0.067 (4)	−0.014 (4)	−0.015 (3)	−0.034 (5)
C53	0.036 (3)	0.078 (4)	0.057 (3)	0.008 (3)	−0.016 (2)	−0.022 (3)
C54	0.038 (2)	0.051 (3)	0.049 (3)	0.014 (2)	−0.013 (2)	−0.025 (2)
C55	0.048 (3)	0.102 (6)	0.084 (5)	0.003 (3)	−0.040 (3)	−0.007 (4)
C56	0.100 (4)	0.109 (5)	0.096 (5)	0.015 (4)	−0.058 (4)	−0.016 (4)
C57	0.082 (5)	0.087 (5)	0.080 (5)	0.017 (4)	−0.050 (4)	−0.005 (4)
C58	0.076 (6)	0.072 (6)	0.065 (6)	0.016 (4)	−0.032 (4)	−0.018 (4)
C59	0.087 (4)	0.052 (3)	0.040 (3)	0.019 (3)	−0.038 (3)	−0.006 (2)
C60	0.083 (4)	0.033 (2)	0.036 (2)	0.006 (2)	−0.030 (2)	−0.0123 (19)
C61	0.102 (5)	0.037 (3)	0.033 (2)	0.004 (3)	−0.026 (3)	−0.009 (2)
C62	0.095 (5)	0.043 (3)	0.030 (2)	−0.006 (3)	−0.012 (3)	−0.008 (2)
C63	0.074 (4)	0.056 (3)	0.044 (3)	0.000 (3)	−0.019 (3)	−0.015 (2)
C64	0.070 (3)	0.038 (2)	0.031 (2)	0.003 (2)	−0.021 (2)	−0.0108 (19)
C65	0.073 (3)	0.028 (2)	0.035 (2)	0.007 (2)	−0.027 (2)	−0.0130 (18)
C66	0.058 (3)	0.065 (3)	0.047 (3)	0.014 (3)	−0.020 (3)	−0.023 (3)
C67	0.070 (4)	0.067 (4)	0.051 (3)	0.027 (3)	−0.029 (3)	−0.028 (3)
C68	0.102 (5)	0.032 (2)	0.043 (3)	0.015 (3)	−0.021 (3)	−0.011 (2)
C69	0.164 (9)	0.069 (4)	0.068 (4)	0.034 (5)	−0.046 (5)	−0.039 (4)
C70	0.084 (6)	0.082 (5)	0.071 (4)	0.012 (4)	−0.016 (4)	−0.030 (4)
C71	0.102 (6)	0.102 (6)	0.106 (6)	0.022 (5)	−0.020 (4)	−0.045 (4)
O13'	0.057 (3)	0.036 (2)	0.039 (4)	0.017 (2)	−0.008 (3)	−0.015 (2)
C10'	0.125 (6)	0.093 (6)	0.093 (5)	−0.013 (4)	−0.024 (4)	−0.069 (5)
C11'	0.096 (5)	0.101 (5)	0.082 (5)	0.017 (4)	−0.034 (4)	−0.066 (4)
C12'	0.122 (6)	0.102 (6)	0.088 (6)	0.012 (4)	−0.038 (4)	−0.049 (4)
C33'	0.058 (3)	0.074 (5)	0.059 (5)	0.005 (3)	−0.033 (3)	−0.003 (3)
C34'	0.076 (5)	0.096 (5)	0.074 (5)	0.004 (4)	−0.036 (4)	−0.013 (4)
C35'	0.071 (5)	0.091 (5)	0.072 (4)	0.012 (4)	−0.037 (4)	−0.016 (4)
C56'	0.100 (4)	0.109 (5)	0.096 (5)	0.015 (4)	−0.058 (4)	−0.016 (4)
C43'	0.053 (5)	0.050 (3)	0.066 (4)	−0.006 (4)	−0.008 (4)	−0.008 (3)
C44'	0.092 (6)	0.072 (4)	0.087 (5)	0.004 (5)	−0.027 (4)	−0.017 (4)
C47'	0.060 (4)	0.044 (3)	0.066 (4)	−0.001 (3)	−0.033 (3)	−0.009 (3)
C48'	0.044 (3)	0.043 (3)	0.044 (3)	0.001 (3)	−0.011 (3)	−0.012 (3)
C57'	0.100 (4)	0.109 (5)	0.096 (5)	0.015 (4)	−0.058 (4)	−0.016 (4)
C58'	0.082 (5)	0.087 (5)	0.080 (5)	0.017 (4)	−0.050 (4)	−0.005 (4)
C70'	0.084 (6)	0.082 (5)	0.071 (4)	0.012 (4)	−0.016 (4)	−0.030 (4)
C71'	0.102 (6)	0.102 (6)	0.106 (6)	0.022 (5)	−0.020 (4)	−0.045 (4)

### Geometric parameters (Å, º)

**Table:**

Cd1—O2	2.219 (3)	C26—C27	1.3900
Cd1—O3	2.236 (3)	C26—C31	1.3900
Cd1—N2	2.277 (6)	C27—C28	1.3900
Cd1—N1	2.312 (5)	C27—H27	0.9500
Cd1—O6	2.335 (4)	C28—C29	1.3900
Cd1—O5	2.456 (3)	C28—H28	0.9500
Cd2—O9	2.206 (3)	C29—C30	1.3900
Cd2—O8	2.237 (3)	C29—H29	0.9500
Cd2—N3	2.282 (5)	C30—C31	1.3900
Cd2—O12	2.308 (4)	C30—C32	1.455 (6)
Cd2—N4	2.315 (5)	C32—H32	0.9500
Cd2—O11	2.444 (3)	C33—C34	1.454 (9)
Cd3—O15	2.181 (4)	C33—C35	1.492 (9)
Cd3—O14	2.206 (3)	C33—H33	1.0000
Cd3—O17	2.259 (4)	C34—H34A	0.9800
Cd3—N6	2.294 (5)	C34—H34B	0.9800
Cd3—N5	2.316 (6)	C34—H34C	0.9800
Na1—O5	2.313 (3)	C35—H35A	0.9900
Na1—O3^i^	2.316 (3)	C35—H35B	0.9900
Na1—O5^i^	2.324 (3)	C36—C37	1.447 (9)
Na1—O2	2.323 (3)	C36—H36	0.9500
Na1—O1	2.449 (3)	C37—C38	1.3900
Na1—O4^i^	2.511 (3)	C37—C42	1.3900
Na2—O11	2.300 (4)	C38—C39	1.3900
Na2—O14	2.347 (4)	C38—H38	0.9500
Na2—O8	2.345 (3)	C39—C40	1.3900
Na2—O18	2.346 (4)	C39—H39	0.9500
Na2—O13'	2.405 (12)	C40—C41	1.3900
Na2—O7	2.475 (4)	C40—H40	0.9500
Na2—O13	2.499 (12)	C41—C42	1.3900
Na3—O15	2.288 (3)	C43—C44	1.494 (10)
Na3—O9	2.301 (4)	C43—H43A	0.9900
Na3—O11	2.305 (3)	C43—H43B	0.9900
Na3—O10	2.377 (5)	C44—H44A	0.9800
Na3—O18	2.376 (5)	C44—H44B	0.9800
Na3—O16	2.477 (4)	C44—H44C	0.9800
O1—C3	1.367 (4)	C45—C46	1.516 (7)
O1—C2	1.439 (7)	C46—H46A	0.9800
O2—C8	1.350 (3)	C46—H46B	0.9800
O3—C19	1.345 (3)	C46—H46C	0.9800
O3—Na1^i^	2.316 (3)	C47—C48	1.488 (9)
O4—C18	1.372 (4)	C47—H47A	0.9800
O4—C20	1.433 (5)	C47—H47B	0.9800
O4—Na1^i^	2.511 (3)	C47—H47C	0.9800
O5—C22	1.263 (6)	C48—H48A	0.9900
O5—Na1^i^	2.324 (3)	C48—H48B	0.9900
O6—C22	1.247 (7)	C49—O13'	1.379 (8)
O7—C26	1.373 (4)	C49—C50	1.3900
O7—C25	1.441 (6)	C49—C54	1.3900
O8—C31	1.350 (4)	C50—C51	1.3900
O9—C42	1.330 (4)	C50—H50	0.9500
O10—C41	1.370 (7)	C51—C52	1.3900
O10—C43'	1.431 (8)	C51—H51	0.9500
O10—C43	1.487 (9)	C52—C53	1.3900
O11—C45	1.266 (6)	C52—H52	0.9500
O12—C45	1.239 (6)	C53—C54	1.3900
O13—C49	1.393 (8)	C53—C55	1.432 (8)
O13—C48	1.466 (9)	C55—H55	0.9500
O14—C54	1.338 (4)	C56—C58	1.488 (10)
O15—C65	1.340 (4)	C56—C57	1.511 (10)
O16—C64	1.374 (4)	C56—H56	1.0000
O16—C66	1.420 (7)	C57—H57A	0.9800
O17—C68	1.266 (7)	C57—H57B	0.9800
O18—C68	1.249 (7)	C57—H57C	0.9800
O19—C70	1.437 (9)	C58—H58A	0.9900
O19—C70'	1.447 (9)	C58—H58B	0.9900
O19—H19	0.8399	C59—C60	1.466 (7)
N1—C9	1.275 (9)	C59—H59	0.9500
N1—C10'	1.464 (9)	C60—C61	1.3900
N1—C10	1.476 (9)	C60—C65	1.3901
N2—C13	1.289 (9)	C61—C62	1.3900
N2—C12	1.462 (9)	C61—H61	0.9500
N2—C11'	1.463 (9)	C62—C63	1.3900
N3—C32	1.288 (8)	C62—H62	0.9500
N3—C33	1.469 (8)	C63—C64	1.3900
N3—C33'	1.502 (9)	C63—H63	0.9500
N4—C36	1.297 (9)	C64—C65	1.3900
N4—C34'	1.455 (9)	C66—C67	1.489 (8)
N4—C35	1.490 (9)	C66—H66A	0.9900
N5—C55	1.270 (9)	C66—H66B	0.9900
N5—C56	1.470 (9)	C67—H67A	0.9800
N5—C56'	1.470 (9)	C67—H67B	0.9800
N6—C59	1.263 (8)	C67—H67C	0.9800
N6—C58'	1.460 (9)	C68—C69	1.513 (8)
N6—C58	1.480 (9)	C69—H69A	0.9800
C1—C2	1.490 (10)	C69—H69B	0.9800
C1—H1A	0.9800	C69—H69C	0.9800
C1—H1B	0.9800	C70—C71	1.493 (10)
C1—H1C	0.9800	C70—H70A	0.9900
C2—H2A	0.9900	C70—H70B	0.9900
C2—H2B	0.9900	C71—H71A	0.9800
C3—C4	1.3900	C71—H71B	0.9800
C3—C8	1.3900	C71—H71C	0.9800
C4—C5	1.3900	O13'—C48'	1.455 (9)
C4—H4	0.9500	C10'—C11'	1.481 (10)
C5—C6	1.3900	C10'—H10B	0.9900
C5—H5	0.9500	C10'—H10C	0.9900
C6—C7	1.3900	C11'—C12'	1.542 (10)
C6—H6	0.9500	C11'—H11'	1.0000
C7—C8	1.3900	C12'—H12C	0.9800
C7—C9	1.464 (8)	C12'—H12D	0.9800
C9—H9	0.9500	C12'—H12E	0.9800
C10—C12	1.493 (10)	C33'—C34'	1.502 (9)
C10—C11	1.517 (10)	C33'—H33B	0.9900
C10—H10	1.0000	C33'—H33C	0.9900
C11—H11A	0.9800	C34'—C35'	1.510 (9)
C11—H11B	0.9800	C34'—H34'	1.0000
C11—H11C	0.9800	C35'—H35C	0.9800
C12—H12A	0.9900	C35'—H35D	0.9800
C12—H12B	0.9900	C35'—H35E	0.9800
C13—C14	1.421 (7)	C56'—C58'	1.491 (10)
C13—H13	0.9500	C56'—C57'	1.504 (10)
C14—C15	1.3900	C56'—H56'	1.0000
C14—C19	1.3900	C43'—C44'	1.461 (10)
C15—C16	1.3900	C43'—H43C	0.9900
C15—H15	0.9500	C43'—H43D	0.9900
C16—C17	1.3900	C44'—H44D	0.9800
C16—H16	0.9500	C44'—H44E	0.9800
C17—C18	1.3900	C44'—H44F	0.9800
C17—H17	0.9500	C47'—C48'	1.505 (9)
C18—C19	1.3900	C47'—H47D	0.9800
C20—C21	1.496 (7)	C47'—H47E	0.9800
C20—H20A	0.9900	C47'—H47F	0.9800
C20—H20B	0.9900	C48'—H48C	0.9900
C21—H21A	0.9800	C48'—H48D	0.9900
C21—H21B	0.9800	C57'—H57D	0.9800
C21—H21C	0.9800	C57'—H57E	0.9800
C22—C23	1.514 (8)	C57'—H57F	0.9800
C23—H23A	0.9800	C58'—H58C	0.9900
C23—H23B	0.9800	C58'—H58D	0.9900
C23—H23C	0.9800	C70'—C71'	1.495 (10)
C24—C25	1.489 (9)	C70'—H70C	0.9900
C24—H24A	0.9800	C70'—H70D	0.9900
C24—H24B	0.9800	C71'—H71D	0.9800
C24—H24C	0.9800	C71'—H71E	0.9800
C25—H25A	0.9900	C71'—H71F	0.9800
C25—H25B	0.9900		
			
O2—Cd1—O3	109.54 (10)	H25A—C25—H25B	108.5
O2—Cd1—N2	145.9 (2)	O7—C26—C27	123.2 (2)
O3—Cd1—N2	79.30 (16)	O7—C26—C31	116.7 (3)
O2—Cd1—N1	79.55 (16)	C27—C26—C31	120.0
O3—Cd1—N1	136.9 (2)	C26—C27—C28	120.0
N2—Cd1—N1	72.9 (2)	C26—C27—H27	120.0
O2—Cd1—O6	107.98 (14)	C28—C27—H27	120.0
O3—Cd1—O6	112.62 (14)	C29—C28—C27	120.0
N2—Cd1—O6	97.7 (2)	C29—C28—H28	120.0
N1—Cd1—O6	103.4 (2)	C27—C28—H28	120.0
O2—Cd1—O5	80.03 (10)	C28—C29—C30	120.0
O3—Cd1—O5	80.08 (10)	C28—C29—H29	120.0
N2—Cd1—O5	133.9 (2)	C30—C29—H29	120.0
N1—Cd1—O5	142.31 (19)	C29—C30—C31	120.0
O6—Cd1—O5	54.44 (12)	C29—C30—C32	113.3 (3)
O9—Cd2—O8	106.76 (12)	C31—C30—C32	126.7 (3)
O9—Cd2—N3	138.91 (17)	O8—C31—C30	123.9 (2)
O8—Cd2—N3	81.96 (14)	O8—C31—C26	116.1 (2)
O9—Cd2—O12	111.44 (13)	C30—C31—C26	120.0
O8—Cd2—O12	109.27 (14)	N3—C32—C30	127.8 (4)
N3—Cd2—O12	102.56 (19)	N3—C32—H32	116.1
O9—Cd2—N4	80.07 (17)	C30—C32—H32	116.1
O8—Cd2—N4	143.63 (18)	C34—C33—N3	115.7 (11)
N3—Cd2—N4	71.44 (17)	C34—C33—C35	109.2 (8)
O12—Cd2—N4	100.6 (2)	N3—C33—C35	105.2 (9)
O9—Cd2—O11	78.18 (11)	C34—C33—H33	108.8
O8—Cd2—O11	79.22 (11)	N3—C33—H33	108.8
N3—Cd2—O11	142.30 (16)	C35—C33—H33	108.8
O12—Cd2—O11	54.89 (11)	C33—C34—H34A	109.5
N4—Cd2—O11	136.42 (17)	C33—C34—H34B	109.5
O15—Cd3—O14	112.82 (12)	H34A—C34—H34B	109.5
O15—Cd3—O17	108.23 (16)	C33—C34—H34C	109.5
O14—Cd3—O17	101.21 (15)	H34A—C34—H34C	109.5
O15—Cd3—N6	80.88 (15)	H34B—C34—H34C	109.5
O14—Cd3—N6	150.75 (18)	C33—C35—N4	108.2 (9)
O17—Cd3—N6	98.34 (19)	C33—C35—H35A	110.1
O15—Cd3—N5	136.80 (19)	N4—C35—H35A	110.1
O14—Cd3—N5	80.76 (16)	C33—C35—H35B	110.1
O17—Cd3—N5	108.8 (2)	N4—C35—H35B	110.1
N6—Cd3—N5	72.4 (2)	H35A—C35—H35B	108.4
O5—Na1—O3^i^	104.14 (12)	N4—C36—C37	128.4 (6)
O5—Na1—O5^i^	86.92 (12)	N4—C36—H36	115.8
O3^i^—Na1—O5^i^	81.32 (11)	C37—C36—H36	115.8
O5—Na1—O2	81.02 (11)	C38—C37—C42	120.0
O3^i^—Na1—O2	173.24 (12)	C38—C37—C36	115.1 (4)
O5^i^—Na1—O2	103.52 (12)	C42—C37—C36	124.9 (4)
O5—Na1—O1	146.28 (13)	C39—C38—C37	120.0
O3^i^—Na1—O1	108.46 (13)	C39—C38—H38	120.0
O5^i^—Na1—O1	106.10 (13)	C37—C38—H38	120.0
O2—Na1—O1	65.85 (12)	C38—C39—C40	120.0
O5—Na1—O4^i^	104.33 (12)	C38—C39—H39	120.0
O3^i^—Na1—O4^i^	65.00 (11)	C40—C39—H39	120.0
O5^i^—Na1—O4^i^	146.09 (12)	C41—C40—C39	120.0
O2—Na1—O4^i^	109.74 (11)	C41—C40—H40	120.0
O1—Na1—O4^i^	82.26 (12)	C39—C40—H40	120.0
O11—Na2—O14	96.85 (14)	O10—C41—C40	123.1 (3)
O11—Na2—O8	80.10 (12)	O10—C41—C42	116.9 (3)
O14—Na2—O8	171.82 (14)	C40—C41—C42	120.0
O11—Na2—O18	89.21 (14)	O9—C42—C41	116.0 (3)
O14—Na2—O18	82.25 (14)	O9—C42—C37	124.0 (3)
O8—Na2—O18	105.19 (14)	C41—C42—C37	120.0
O11—Na2—O13'	100.4 (3)	O10—C43—C44	119.0 (11)
O14—Na2—O13'	67.1 (2)	O10—C43—H43A	107.6
O8—Na2—O13'	105.8 (2)	C44—C43—H43A	107.6
O18—Na2—O13'	148.7 (2)	O10—C43—H43B	107.6
O11—Na2—O7	144.82 (13)	C44—C43—H43B	107.6
O14—Na2—O7	116.68 (15)	H43A—C43—H43B	107.0
O8—Na2—O7	65.21 (12)	C43—C44—H44A	109.5
O18—Na2—O7	104.81 (14)	C43—C44—H44B	109.5
O13'—Na2—O7	84.3 (4)	H44A—C44—H44B	109.5
O11—Na2—O13	92.8 (3)	C43—C44—H44C	109.5
O14—Na2—O13	65.4 (2)	H44A—C44—H44C	109.5
O8—Na2—O13	107.0 (2)	H44B—C44—H44C	109.5
O18—Na2—O13	147.6 (2)	O12—C45—O11	122.3 (4)
O13'—Na2—O13	7.9 (6)	O12—C45—C46	120.0 (5)
O7—Na2—O13	91.9 (3)	O11—C45—C46	117.7 (5)
O15—Na3—O9	167.48 (16)	C45—C46—H46A	109.5
O15—Na3—O11	104.39 (14)	C45—C46—H46B	109.5
O9—Na3—O11	79.27 (12)	H46A—C46—H46B	109.5
O15—Na3—O10	107.70 (16)	C45—C46—H46C	109.5
O9—Na3—O10	67.33 (15)	H46A—C46—H46C	109.5
O11—Na3—O10	146.47 (17)	H46B—C46—H46C	109.5
O15—Na3—O18	83.33 (14)	C48—C47—H47A	109.5
O9—Na3—O18	108.90 (14)	C48—C47—H47B	109.5
O11—Na3—O18	88.35 (14)	H47A—C47—H47B	109.5
O10—Na3—O18	104.68 (16)	C48—C47—H47C	109.5
O15—Na3—O16	65.39 (13)	H47A—C47—H47C	109.5
O9—Na3—O16	102.18 (14)	H47B—C47—H47C	109.5
O11—Na3—O16	103.52 (14)	O13—C48—C47	117.1 (10)
O10—Na3—O16	81.57 (16)	O13—C48—H48A	108.0
O18—Na3—O16	148.33 (14)	C47—C48—H48A	108.0
C3—O1—C2	118.9 (4)	O13—C48—H48B	108.0
C3—O1—Na1	109.8 (2)	C47—C48—H48B	108.0
C2—O1—Na1	127.3 (3)	H48A—C48—H48B	107.3
C8—O2—Cd1	133.1 (2)	O13'—C49—C50	121.8 (5)
C8—O2—Na1	115.0 (2)	O13'—C49—C54	117.9 (5)
Cd1—O2—Na1	102.00 (11)	C50—C49—C54	120.0
C19—O3—Cd1	132.7 (2)	C50—C49—O13	122.5 (5)
C19—O3—Na1^i^	113.3 (2)	C54—C49—O13	116.7 (6)
Cd1—O3—Na1^i^	101.80 (12)	C51—C50—C49	120.0
C18—O4—C20	117.5 (3)	C51—C50—H50	120.0
C18—O4—Na1^i^	106.3 (2)	C49—C50—H50	120.0
C20—O4—Na1^i^	123.6 (3)	C50—C51—C52	120.0
C22—O5—Na1	129.6 (3)	C50—C51—H51	120.0
C22—O5—Na1^i^	136.6 (3)	C52—C51—H51	120.0
Na1—O5—Na1^i^	93.08 (12)	C53—C52—C51	120.0
C22—O5—Cd1	88.8 (3)	C53—C52—H52	120.0
Na1—O5—Cd1	95.49 (11)	C51—C52—H52	120.0
Na1^i^—O5—Cd1	95.24 (11)	C52—C53—C54	120.0
C22—O6—Cd1	94.8 (3)	C52—C53—C55	113.5 (4)
C26—O7—C25	117.3 (4)	C54—C53—C55	126.3 (4)
C26—O7—Na2	111.2 (2)	O14—C54—C53	123.8 (3)
C25—O7—Na2	121.0 (3)	O14—C54—C49	116.2 (3)
C31—O8—Cd2	130.8 (2)	C53—C54—C49	120.0
C31—O8—Na2	116.2 (2)	N5—C55—C53	127.9 (5)
Cd2—O8—Na2	102.36 (13)	N5—C55—H55	116.0
C42—O9—Cd2	134.6 (3)	C53—C55—H55	116.0
C42—O9—Na3	114.2 (3)	N5—C56—C58	114.8 (14)
Cd2—O9—Na3	104.02 (13)	N5—C56—C57	105.4 (13)
C41—O10—C43'	104.3 (6)	C58—C56—C57	103.4 (8)
C41—O10—C43	127.9 (6)	N5—C56—H56	111.0
C41—O10—Na3	109.9 (3)	C58—C56—H56	111.0
C43'—O10—Na3	137.9 (7)	C57—C56—H56	111.0
C43—O10—Na3	117.8 (7)	C56—C57—H57A	109.5
C45—O11—Na2	129.8 (3)	C56—C57—H57B	109.5
C45—O11—Na3	136.4 (3)	H57A—C57—H57B	109.5
Na2—O11—Na3	92.66 (13)	C56—C57—H57C	109.5
C45—O11—Cd2	87.9 (3)	H57A—C57—H57C	109.5
Na2—O11—Cd2	97.62 (12)	H57B—C57—H57C	109.5
Na3—O11—Cd2	96.82 (12)	N6—C58—C56	102.1 (11)
C45—O12—Cd2	94.9 (3)	N6—C58—H58A	111.4
C49—O13—C48	136.3 (10)	C56—C58—H58A	111.4
C49—O13—Na2	109.7 (6)	N6—C58—H58B	111.4
C48—O13—Na2	113.1 (7)	C56—C58—H58B	111.4
C54—O14—Cd3	132.8 (3)	H58A—C58—H58B	109.2
C54—O14—Na2	119.3 (3)	N6—C59—C60	127.5 (4)
Cd3—O14—Na2	106.76 (15)	N6—C59—H59	116.3
C65—O15—Cd3	133.5 (2)	C60—C59—H59	116.3
C65—O15—Na3	116.6 (3)	C61—C60—C65	120.0
Cd3—O15—Na3	105.86 (14)	C61—C60—C59	115.2 (3)
C64—O16—C66	118.8 (4)	C65—C60—C59	124.5 (3)
C64—O16—Na3	107.6 (2)	C60—C61—C62	120.0
C66—O16—Na3	128.9 (3)	C60—C61—H61	120.0
C68—O17—Cd3	104.0 (3)	C62—C61—H61	120.0
C68—O18—Na2	129.0 (4)	C63—C62—C61	120.0
C68—O18—Na3	140.2 (4)	C63—C62—H62	120.0
Na2—O18—Na3	89.70 (14)	C61—C62—H62	120.0
C70—O19—H19	120.5	C62—C63—C64	120.0
C70'—O19—H19	119.3	C62—C63—H63	120.0
C9—N1—C10'	112.9 (8)	C64—C63—H63	120.0
C9—N1—C10	121.5 (8)	O16—C64—C63	124.0 (3)
C9—N1—Cd1	129.5 (4)	O16—C64—C65	115.9 (3)
C10'—N1—Cd1	115.8 (8)	C63—C64—C65	120.0
C10—N1—Cd1	108.9 (7)	O15—C65—C64	115.8 (3)
C13—N2—C12	120.6 (10)	O15—C65—C60	124.1 (3)
C13—N2—C11'	116.0 (8)	C64—C65—C60	120.0
C13—N2—Cd1	131.3 (4)	O16—C66—C67	108.1 (5)
C12—N2—Cd1	106.7 (10)	O16—C66—H66A	110.1
C11'—N2—Cd1	112.2 (7)	C67—C66—H66A	110.1
C32—N3—C33	113.8 (6)	O16—C66—H66B	110.1
C32—N3—C33'	123.9 (7)	C67—C66—H66B	110.1
C32—N3—Cd2	127.6 (4)	H66A—C66—H66B	108.4
C33—N3—Cd2	118.0 (5)	C66—C67—H67A	109.5
C33'—N3—Cd2	105.8 (6)	C66—C67—H67B	109.5
C36—N4—C34'	111.0 (7)	H67A—C67—H67B	109.5
C36—N4—C35	126.7 (8)	C66—C67—H67C	109.5
C36—N4—Cd2	127.9 (5)	H67A—C67—H67C	109.5
C34'—N4—Cd2	115.8 (6)	H67B—C67—H67C	109.5
C35—N4—Cd2	105.3 (6)	O18—C68—O17	122.4 (5)
C55—N5—C56	121.7 (9)	O18—C68—C69	120.0 (6)
C55—N5—C56'	112.4 (9)	O17—C68—C69	117.6 (6)
C55—N5—Cd3	127.9 (4)	C68—C69—H69A	109.5
C56—N5—Cd3	110.3 (8)	C68—C69—H69B	109.5
C56'—N5—Cd3	118.9 (8)	H69A—C69—H69B	109.5
C59—N6—C58'	117.5 (9)	C68—C69—H69C	109.5
C59—N6—C58	123.9 (8)	H69A—C69—H69C	109.5
C59—N6—Cd3	128.9 (4)	H69B—C69—H69C	109.5
C58'—N6—Cd3	112.2 (8)	O19—C70—C71	103.9 (13)
C58—N6—Cd3	106.8 (8)	O19—C70—H70A	111.0
C2—C1—H1A	109.5	C71—C70—H70A	111.0
C2—C1—H1B	109.5	O19—C70—H70B	111.0
H1A—C1—H1B	109.5	C71—C70—H70B	111.0
C2—C1—H1C	109.5	H70A—C70—H70B	109.0
H1A—C1—H1C	109.5	C70—C71—H71A	109.5
H1B—C1—H1C	109.5	C70—C71—H71B	109.5
O1—C2—C1	108.6 (5)	H71A—C71—H71B	109.5
O1—C2—H2A	110.0	C70—C71—H71C	109.5
C1—C2—H2A	110.0	H71A—C71—H71C	109.5
O1—C2—H2B	110.0	H71B—C71—H71C	109.5
C1—C2—H2B	110.0	C49—O13'—C48'	107.8 (8)
H2A—C2—H2B	108.4	C49—O13'—Na2	115.5 (6)
O1—C3—C4	123.9 (2)	C48'—O13'—Na2	136.7 (6)
O1—C3—C8	116.1 (2)	N1—C10'—C11'	107.7 (11)
C4—C3—C8	120.0	N1—C10'—H10B	110.2
C5—C4—C3	120.0	C11'—C10'—H10B	110.2
C5—C4—H4	120.0	N1—C10'—H10C	110.2
C3—C4—H4	120.0	C11'—C10'—H10C	110.2
C4—C5—C6	120.0	H10B—C10'—H10C	108.5
C4—C5—H5	120.0	N2—C11'—C10'	112.5 (13)
C6—C5—H5	120.0	N2—C11'—C12'	105.0 (11)
C7—C6—C5	120.0	C10'—C11'—C12'	102.1 (8)
C7—C6—H6	120.0	N2—C11'—H11'	112.2
C5—C6—H6	120.0	C10'—C11'—H11'	112.2
C8—C7—C6	120.0	C12'—C11'—H11'	112.2
C8—C7—C9	125.6 (3)	C11'—C12'—H12C	109.5
C6—C7—C9	114.3 (3)	C11'—C12'—H12D	109.5
O2—C8—C7	123.4 (2)	H12C—C12'—H12D	109.5
O2—C8—C3	116.5 (2)	C11'—C12'—H12E	109.5
C7—C8—C3	120.0	H12C—C12'—H12E	109.5
N1—C9—C7	126.5 (4)	H12D—C12'—H12E	109.5
N1—C9—H9	116.7	N3—C33'—C34'	104.8 (9)
C7—C9—H9	116.7	N3—C33'—H33B	110.8
N1—C10—C12	114.9 (14)	C34'—C33'—H33B	110.8
N1—C10—C11	103.9 (11)	N3—C33'—H33C	110.8
C12—C10—C11	103.6 (9)	C34'—C33'—H33C	110.8
N1—C10—H10	111.3	H33B—C33'—H33C	108.9
C12—C10—H10	111.3	N4—C34'—C33'	104.2 (9)
C11—C10—H10	111.3	N4—C34'—C35'	123.5 (12)
C10—C11—H11A	109.5	C33'—C34'—C35'	103.1 (8)
C10—C11—H11B	109.5	N4—C34'—H34'	108.4
H11A—C11—H11B	109.5	C33'—C34'—H34'	108.4
C10—C11—H11C	109.5	C35'—C34'—H34'	108.4
H11A—C11—H11C	109.5	C34'—C35'—H35C	109.5
H11B—C11—H11C	109.5	C34'—C35'—H35D	109.5
N2—C12—C10	101.2 (11)	H35C—C35'—H35D	109.5
N2—C12—H12A	111.5	C34'—C35'—H35E	109.5
C10—C12—H12A	111.5	H35C—C35'—H35E	109.5
N2—C12—H12B	111.5	H35D—C35'—H35E	109.5
C10—C12—H12B	111.5	N5—C56'—C58'	107.0 (13)
H12A—C12—H12B	109.3	N5—C56'—C57'	119.7 (16)
N2—C13—C14	125.6 (5)	C58'—C56'—C57'	102.1 (8)
N2—C13—H13	117.2	N5—C56'—H56'	109.2
C14—C13—H13	117.2	C58'—C56'—H56'	109.2
C15—C14—C19	120.0	C57'—C56'—H56'	109.2
C15—C14—C13	113.1 (3)	O10—C43'—C44'	97.4 (11)
C19—C14—C13	126.8 (3)	O10—C43'—H43C	112.3
C14—C15—C16	120.0	C44'—C43'—H43C	112.3
C14—C15—H15	120.0	O10—C43'—H43D	112.3
C16—C15—H15	120.0	C44'—C43'—H43D	112.3
C17—C16—C15	120.0	H43C—C43'—H43D	109.9
C17—C16—H16	120.0	C43'—C44'—H44D	109.5
C15—C16—H16	120.0	C43'—C44'—H44E	109.5
C16—C17—C18	120.0	H44D—C44'—H44E	109.5
C16—C17—H17	120.0	C43'—C44'—H44F	109.5
C18—C17—H17	120.0	H44D—C44'—H44F	109.5
O4—C18—C19	116.2 (2)	H44E—C44'—H44F	109.5
O4—C18—C17	123.8 (2)	C48'—C47'—H47D	109.5
C19—C18—C17	120.0	C48'—C47'—H47E	109.5
O3—C19—C18	116.6 (2)	H47D—C47'—H47E	109.5
O3—C19—C14	123.3 (2)	C48'—C47'—H47F	109.5
C18—C19—C14	120.0	H47D—C47'—H47F	109.5
O4—C20—C21	108.4 (4)	H47E—C47'—H47F	109.5
O4—C20—H20A	110.0	O13'—C48'—C47'	104.4 (8)
C21—C20—H20A	110.0	O13'—C48'—H48C	110.9
O4—C20—H20B	110.0	C47'—C48'—H48C	110.9
C21—C20—H20B	110.0	O13'—C48'—H48D	110.9
H20A—C20—H20B	108.4	C47'—C48'—H48D	110.9
C20—C21—H21A	109.5	H48C—C48'—H48D	108.9
C20—C21—H21B	109.5	C56'—C57'—H57D	109.5
H21A—C21—H21B	109.5	C56'—C57'—H57E	109.5
C20—C21—H21C	109.5	H57D—C57'—H57E	109.5
H21A—C21—H21C	109.5	C56'—C57'—H57F	109.5
H21B—C21—H21C	109.5	H57D—C57'—H57F	109.5
O6—C22—O5	121.9 (4)	H57E—C57'—H57F	109.5
O6—C22—C23	120.1 (6)	N6—C58'—C56'	115.9 (13)
O5—C22—C23	118.0 (6)	N6—C58'—H58C	108.3
C22—C23—H23A	109.5	C56'—C58'—H58C	108.3
C22—C23—H23B	109.5	N6—C58'—H58D	108.3
H23A—C23—H23B	109.5	C56'—C58'—H58D	108.3
C22—C23—H23C	109.5	H58C—C58'—H58D	107.4
H23A—C23—H23C	109.5	O19—C70'—C71'	111.0 (13)
H23B—C23—H23C	109.5	O19—C70'—H70C	109.4
C25—C24—H24A	109.5	C71'—C70'—H70C	109.4
C25—C24—H24B	109.5	O19—C70'—H70D	109.4
H24A—C24—H24B	109.5	C71'—C70'—H70D	109.4
C25—C24—H24C	109.5	H70C—C70'—H70D	108.0
H24A—C24—H24C	109.5	C70'—C71'—H71D	109.5
H24B—C24—H24C	109.5	C70'—C71'—H71E	109.5
O7—C25—C24	107.7 (5)	H71D—C71'—H71E	109.5
O7—C25—H25A	110.2	C70'—C71'—H71F	109.5
C24—C25—H25A	110.2	H71D—C71'—H71F	109.5
O7—C25—H25B	110.2	H71E—C71'—H71F	109.5
C24—C25—H25B	110.2		
			
O5—Na1—O1—C3	−21.7 (4)	O8—Cd2—N4—C34'	−46.1 (9)
O3^i^—Na1—O1—C3	142.8 (2)	N3—Cd2—N4—C34'	−0.9 (8)
O5^i^—Na1—O1—C3	−131.1 (2)	O12—Cd2—N4—C34'	98.9 (8)
O2—Na1—O1—C3	−33.2 (2)	O11—Cd2—N4—C34'	148.1 (7)
O4^i^—Na1—O1—C3	82.5 (3)	O9—Cd2—N4—C35	175.7 (6)
O5—Na1—O1—C2	−178.6 (4)	O8—Cd2—N4—C35	−79.5 (7)
O3^i^—Na1—O1—C2	−14.1 (4)	N3—Cd2—N4—C35	−34.4 (6)
O5^i^—Na1—O1—C2	72.0 (4)	O12—Cd2—N4—C35	65.5 (6)
O2—Na1—O1—C2	169.9 (4)	O11—Cd2—N4—C35	114.7 (5)
O4^i^—Na1—O1—C2	−74.4 (4)	O15—Cd3—N5—C55	−117.5 (7)
O3—Cd1—O2—C8	−151.3 (3)	O14—Cd3—N5—C55	−3.8 (8)
N2—Cd1—O2—C8	−51.3 (5)	O17—Cd3—N5—C55	95.0 (8)
N1—Cd1—O2—C8	−15.1 (3)	N6—Cd3—N5—C55	−172.0 (8)
O6—Cd1—O2—C8	85.7 (3)	O15—Cd3—N5—C56	65.9 (10)
O5—Cd1—O2—C8	133.1 (3)	O14—Cd3—N5—C56	179.5 (9)
O3—Cd1—O2—Na1	66.45 (14)	O17—Cd3—N5—C56	−81.7 (9)
N2—Cd1—O2—Na1	166.4 (3)	N6—Cd3—N5—C56	11.3 (9)
N1—Cd1—O2—Na1	−157.3 (2)	O15—Cd3—N5—C56'	52.1 (11)
O6—Cd1—O2—Na1	−56.53 (15)	O14—Cd3—N5—C56'	165.7 (11)
O5—Cd1—O2—Na1	−9.20 (11)	O17—Cd3—N5—C56'	−95.5 (11)
O5—Na1—O2—C8	−140.7 (2)	N6—Cd3—N5—C56'	−2.4 (10)
O5^i^—Na1—O2—C8	134.7 (2)	O15—Cd3—N6—C59	1.6 (6)
O1—Na1—O2—C8	32.8 (2)	O14—Cd3—N6—C59	122.7 (6)
O4^i^—Na1—O2—C8	−38.6 (3)	O17—Cd3—N6—C59	−105.7 (6)
O5—Na1—O2—Cd1	9.74 (12)	N5—Cd3—N6—C59	147.2 (7)
O5^i^—Na1—O2—Cd1	−74.87 (14)	O15—Cd3—N6—C58'	−164.4 (8)
O1—Na1—O2—Cd1	−176.69 (15)	O14—Cd3—N6—C58'	−43.3 (9)
O4^i^—Na1—O2—Cd1	111.84 (12)	O17—Cd3—N6—C58'	88.3 (8)
O2—Cd1—O3—C19	156.1 (3)	N5—Cd3—N6—C58'	−18.8 (8)
N2—Cd1—O3—C19	10.2 (4)	O15—Cd3—N6—C58	174.0 (7)
N1—Cd1—O3—C19	60.5 (4)	O14—Cd3—N6—C58	−64.9 (8)
O6—Cd1—O3—C19	−83.7 (3)	O17—Cd3—N6—C58	66.7 (7)
O5—Cd1—O3—C19	−128.3 (3)	N5—Cd3—N6—C58	−40.4 (6)
O2—Cd1—O3—Na1^i^	−66.05 (14)	C3—O1—C2—C1	166.2 (4)
N2—Cd1—O3—Na1^i^	148.1 (2)	Na1—O1—C2—C1	−38.7 (6)
N1—Cd1—O3—Na1^i^	−161.6 (2)	C2—O1—C3—C4	9.2 (5)
O6—Cd1—O3—Na1^i^	54.13 (16)	Na1—O1—C3—C4	−149.98 (19)
O5—Cd1—O3—Na1^i^	9.56 (12)	C2—O1—C3—C8	−168.8 (4)
O3^i^—Na1—O5—C22	−91.2 (4)	Na1—O1—C3—C8	32.0 (3)
O5^i^—Na1—O5—C22	−171.5 (4)	O1—C3—C4—C5	−177.9 (3)
O2—Na1—O5—C22	84.3 (4)	C8—C3—C4—C5	0.0
O1—Na1—O5—C22	73.7 (5)	C3—C4—C5—C6	0.0
O4^i^—Na1—O5—C22	−23.9 (4)	C4—C5—C6—C7	0.0
O3^i^—Na1—O5—Na1^i^	80.24 (12)	C5—C6—C7—C8	0.0
O5^i^—Na1—O5—Na1^i^	0.0	C5—C6—C7—C9	176.4 (4)
O2—Na1—O5—Na1^i^	−104.21 (12)	Cd1—O2—C8—C7	10.1 (4)
O1—Na1—O5—Na1^i^	−114.8 (2)	Na1—O2—C8—C7	148.72 (19)
O4^i^—Na1—O5—Na1^i^	147.57 (12)	Cd1—O2—C8—C3	−168.0 (2)
O3^i^—Na1—O5—Cd1	175.82 (10)	Na1—O2—C8—C3	−29.3 (3)
O5^i^—Na1—O5—Cd1	95.57 (12)	C6—C7—C8—O2	−178.0 (3)
O2—Na1—O5—Cd1	−8.64 (10)	C9—C7—C8—O2	6.0 (5)
O1—Na1—O5—Cd1	−19.3 (3)	C6—C7—C8—C3	0.0
O4^i^—Na1—O5—Cd1	−116.86 (11)	C9—C7—C8—C3	−176.0 (5)
O2—Cd1—O5—C22	−120.6 (3)	O1—C3—C8—O2	−3.8 (3)
O3—Cd1—O5—C22	127.4 (3)	C4—C3—C8—O2	178.1 (3)
N2—Cd1—O5—C22	62.8 (4)	O1—C3—C8—C7	178.1 (3)
N1—Cd1—O5—C22	−62.5 (4)	C4—C3—C8—C7	0.0
O6—Cd1—O5—C22	0.2 (3)	C10'—N1—C9—C7	160.2 (10)
O2—Cd1—O5—Na1	9.08 (11)	C10—N1—C9—C7	178.7 (9)
O3—Cd1—O5—Na1	−102.97 (12)	Cd1—N1—C9—C7	−3.7 (12)
N2—Cd1—O5—Na1	−167.5 (2)	C8—C7—C9—N1	−9.0 (10)
N1—Cd1—O5—Na1	67.2 (3)	C6—C7—C9—N1	174.8 (7)
O6—Cd1—O5—Na1	129.79 (19)	C9—N1—C10—C12	157.2 (10)
O2—Cd1—O5—Na1^i^	102.69 (12)	C10'—N1—C10—C12	−136 (5)
O3—Cd1—O5—Na1^i^	−9.36 (11)	Cd1—N1—C10—C12	−20.9 (13)
N2—Cd1—O5—Na1^i^	−73.9 (3)	C9—N1—C10—C11	−90.4 (12)
N1—Cd1—O5—Na1^i^	160.8 (3)	C10'—N1—C10—C11	−24 (4)
O6—Cd1—O5—Na1^i^	−136.60 (19)	Cd1—N1—C10—C11	91.5 (10)
O2—Cd1—O6—C22	62.7 (3)	C13—N2—C12—C10	130.0 (12)
O3—Cd1—O6—C22	−58.3 (4)	C11'—N2—C12—C10	46 (2)
N2—Cd1—O6—C22	−139.9 (4)	Cd1—N2—C12—C10	−62.4 (11)
N1—Cd1—O6—C22	145.9 (3)	N1—C10—C12—N2	55.6 (14)
O5—Cd1—O6—C22	−0.2 (3)	C11—C10—C12—N2	−57.0 (15)
O11—Na2—O7—C26	19.7 (4)	C12—N2—C13—C14	164.0 (9)
O14—Na2—O7—C26	−141.1 (2)	C11'—N2—C13—C14	−170.8 (9)
O8—Na2—O7—C26	30.1 (2)	Cd1—N2—C13—C14	−0.1 (14)
O18—Na2—O7—C26	130.3 (3)	N2—C13—C14—C15	−174.4 (8)
O13'—Na2—O7—C26	−80.2 (3)	N2—C13—C14—C19	7.7 (12)
O13—Na2—O7—C26	−77.8 (3)	C19—C14—C15—C16	0.0
O11—Na2—O7—C25	163.6 (3)	C13—C14—C15—C16	−178.1 (5)
O14—Na2—O7—C25	2.7 (4)	C14—C15—C16—C17	0.0
O8—Na2—O7—C25	173.9 (4)	C15—C16—C17—C18	0.0
O18—Na2—O7—C25	−85.9 (4)	C20—O4—C18—C19	−177.5 (3)
O13'—Na2—O7—C25	63.6 (4)	Na1^i^—O4—C18—C19	−34.2 (3)
O13—Na2—O7—C25	66.0 (4)	C20—O4—C18—C17	3.0 (5)
O9—Cd2—O8—C31	150.4 (3)	Na1^i^—O4—C18—C17	146.30 (18)
N3—Cd2—O8—C31	11.6 (3)	C16—C17—C18—O4	179.5 (3)
O12—Cd2—O8—C31	−88.9 (3)	C16—C17—C18—C19	0.0
N4—Cd2—O8—C31	54.4 (4)	Cd1—O3—C19—C18	170.7 (2)
O11—Cd2—O8—C31	−135.6 (3)	Na1^i^—O3—C19—C18	36.4 (3)
O9—Cd2—O8—Na2	−67.66 (14)	Cd1—O3—C19—C14	−7.8 (4)
N3—Cd2—O8—Na2	153.5 (2)	Na1^i^—O3—C19—C14	−142.12 (19)
O12—Cd2—O8—Na2	52.99 (15)	O4—C18—C19—O3	1.9 (3)
N4—Cd2—O8—Na2	−163.7 (3)	C17—C18—C19—O3	−178.5 (3)
O11—Cd2—O8—Na2	6.37 (12)	O4—C18—C19—C14	−179.5 (3)
O11—Na2—O8—C31	141.9 (3)	C17—C18—C19—C14	0.0
O18—Na2—O8—C31	−131.7 (3)	C15—C14—C19—O3	178.4 (3)
O13'—Na2—O8—C31	43.8 (4)	C13—C14—C19—O3	−3.7 (6)
O7—Na2—O8—C31	−32.1 (2)	C15—C14—C19—C18	0.0
O13—Na2—O8—C31	52.0 (4)	C13—C14—C19—C18	177.9 (5)
O11—Na2—O8—Cd2	−6.76 (12)	C18—O4—C20—C21	−177.1 (3)
O18—Na2—O8—Cd2	79.70 (16)	Na1^i^—O4—C20—C21	46.4 (5)
O13'—Na2—O8—Cd2	−104.9 (4)	Cd1—O6—C22—O5	0.3 (5)
O7—Na2—O8—Cd2	179.28 (16)	Cd1—O6—C22—C23	−178.8 (5)
O13—Na2—O8—Cd2	−96.7 (3)	Na1—O5—C22—O6	−96.4 (6)
O8—Cd2—O9—C42	−148.3 (3)	Na1^i^—O5—C22—O6	96.1 (6)
N3—Cd2—O9—C42	−51.2 (5)	Cd1—O5—C22—O6	−0.3 (5)
O12—Cd2—O9—C42	92.5 (4)	Na1—O5—C22—C23	82.7 (6)
N4—Cd2—O9—C42	−5.1 (4)	Na1^i^—O5—C22—C23	−84.8 (6)
O11—Cd2—O9—C42	137.0 (4)	Cd1—O5—C22—C23	178.8 (5)
O8—Cd2—O9—Na3	64.69 (15)	C26—O7—C25—C24	179.2 (4)
N3—Cd2—O9—Na3	161.7 (2)	Na2—O7—C25—C24	37.5 (6)
O12—Cd2—O9—Na3	−54.56 (18)	C25—O7—C26—C27	4.5 (5)
N4—Cd2—O9—Na3	−152.1 (2)	Na2—O7—C26—C27	149.8 (2)
O11—Cd2—O9—Na3	−10.08 (13)	C25—O7—C26—C31	−172.5 (3)
O15—Na3—O9—C42	−36.0 (8)	Na2—O7—C26—C31	−27.2 (3)
O11—Na3—O9—C42	−144.2 (3)	O7—C26—C27—C28	−176.9 (4)
O10—Na3—O9—C42	32.8 (3)	C31—C26—C27—C28	0.0
O18—Na3—O9—C42	131.3 (3)	C26—C27—C28—C29	0.0
O16—Na3—O9—C42	−42.5 (3)	C27—C28—C29—C30	0.0
O15—Na3—O9—Cd2	118.9 (6)	C28—C29—C30—C31	0.0
O11—Na3—O9—Cd2	10.66 (14)	C28—C29—C30—C32	179.0 (4)
O10—Na3—O9—Cd2	−172.30 (19)	Cd2—O8—C31—C30	−8.8 (4)
O18—Na3—O9—Cd2	−73.83 (17)	Na2—O8—C31—C30	−146.6 (2)
O16—Na3—O9—Cd2	112.35 (15)	Cd2—O8—C31—C26	168.7 (2)
O15—Na3—O10—C41	136.5 (3)	Na2—O8—C31—C26	30.9 (3)
O9—Na3—O10—C41	−31.2 (3)	C29—C30—C31—O8	177.4 (3)
O11—Na3—O10—C41	−26.0 (5)	C32—C30—C31—O8	−1.5 (5)
O18—Na3—O10—C41	−135.9 (3)	C29—C30—C31—C26	0.0
O16—Na3—O10—C41	75.8 (3)	C32—C30—C31—C26	−178.9 (5)
O15—Na3—O10—C43'	−6.0 (10)	O7—C26—C31—O8	−0.5 (4)
O9—Na3—O10—C43'	−173.7 (10)	C27—C26—C31—O8	−177.6 (3)
O11—Na3—O10—C43'	−168.4 (10)	O7—C26—C31—C30	177.1 (3)
O18—Na3—O10—C43'	81.6 (10)	C27—C26—C31—C30	0.0
O16—Na3—O10—C43'	−66.7 (10)	C33—N3—C32—C30	−165.7 (8)
O15—Na3—O10—C43	−21.8 (8)	C33'—N3—C32—C30	164.0 (7)
O9—Na3—O10—C43	170.4 (8)	Cd2—N3—C32—C30	5.4 (11)
O11—Na3—O10—C43	175.7 (8)	C29—C30—C32—N3	−175.8 (6)
O18—Na3—O10—C43	65.7 (8)	C31—C30—C32—N3	3.1 (10)
O16—Na3—O10—C43	−82.5 (8)	C32—N3—C33—C34	74.7 (12)
O14—Na2—O11—C45	84.8 (4)	C33'—N3—C33—C34	−167 (2)
O8—Na2—O11—C45	−87.5 (4)	Cd2—N3—C33—C34	−97.3 (10)
O18—Na2—O11—C45	166.9 (4)	C32—N3—C33—C35	−164.7 (8)
O13'—Na2—O11—C45	16.9 (4)	C33'—N3—C33—C35	−46.9 (13)
O7—Na2—O11—C45	−78.0 (5)	Cd2—N3—C33—C35	23.2 (11)
O13—Na2—O11—C45	19.3 (4)	C34—C33—C35—N4	69.7 (13)
O14—Na2—O11—Na3	−84.33 (13)	N3—C33—C35—N4	−55.1 (12)
O8—Na2—O11—Na3	103.34 (12)	C36—N4—C35—C33	−123.9 (10)
O18—Na2—O11—Na3	−2.23 (14)	C34'—N4—C35—C33	−53.0 (12)
O13'—Na2—O11—Na3	−152.3 (2)	Cd2—N4—C35—C33	61.1 (9)
O7—Na2—O11—Na3	112.9 (2)	C34'—N4—C36—C37	155.9 (9)
O13—Na2—O11—Na3	−149.9 (2)	C35—N4—C36—C37	−170.7 (8)
O14—Na2—O11—Cd2	178.42 (12)	Cd2—N4—C36—C37	3.1 (12)
O8—Na2—O11—Cd2	6.09 (11)	N4—C36—C37—C38	176.1 (7)
O18—Na2—O11—Cd2	−99.48 (13)	N4—C36—C37—C42	−4.6 (11)
O13'—Na2—O11—Cd2	110.5 (2)	C42—C37—C38—C39	0.0
O7—Na2—O11—Cd2	15.6 (3)	C36—C37—C38—C39	179.4 (5)
O13—Na2—O11—Cd2	112.9 (2)	C37—C38—C39—C40	0.0
O15—Na3—O11—C45	−83.0 (5)	C38—C39—C40—C41	0.0
O9—Na3—O11—C45	84.8 (4)	C43'—O10—C41—C40	3.1 (9)
O10—Na3—O11—C45	79.8 (5)	C43—O10—C41—C40	3.6 (11)
O18—Na3—O11—C45	−165.6 (4)	Na3—O10—C41—C40	−152.0 (2)
O16—Na3—O11—C45	−15.3 (5)	C43'—O10—C41—C42	−176.2 (8)
O15—Na3—O11—Na2	84.88 (15)	C43—O10—C41—C42	−175.7 (9)
O9—Na3—O11—Na2	−107.39 (13)	Na3—O10—C41—C42	28.7 (4)
O10—Na3—O11—Na2	−112.3 (3)	C39—C40—C41—O10	−179.3 (5)
O18—Na3—O11—Na2	2.20 (13)	C39—C40—C41—C42	0.0
O16—Na3—O11—Na2	152.51 (13)	Cd2—O9—C42—C41	−175.0 (3)
O15—Na3—O11—Cd2	−177.12 (13)	Na3—O9—C42—C41	−30.4 (4)
O9—Na3—O11—Cd2	−9.39 (12)	Cd2—O9—C42—C37	5.4 (5)
O10—Na3—O11—Cd2	−14.3 (3)	Na3—O9—C42—C37	150.1 (3)
O18—Na3—O11—Cd2	100.21 (14)	O10—C41—C42—O9	−0.3 (5)
O16—Na3—O11—Cd2	−109.48 (13)	C40—C41—C42—O9	−179.6 (4)
O9—Cd2—O11—C45	−126.7 (3)	O10—C41—C42—C37	179.3 (4)
O8—Cd2—O11—C45	123.5 (3)	C40—C41—C42—C37	0.0
N3—Cd2—O11—C45	62.1 (4)	C38—C37—C42—O9	179.5 (4)
O12—Cd2—O11—C45	0.5 (3)	C36—C37—C42—O9	0.2 (6)
N4—Cd2—O11—C45	−65.1 (4)	C38—C37—C42—C41	0.0
O9—Cd2—O11—Na2	103.45 (14)	C36—C37—C42—C41	−179.3 (5)
O8—Cd2—O11—Na2	−6.40 (12)	C41—O10—C43—C44	166.7 (12)
N3—Cd2—O11—Na2	−67.8 (3)	C43'—O10—C43—C44	168 (4)
O12—Cd2—O11—Na2	−129.40 (19)	Na3—O10—C43—C44	−39.4 (18)
N4—Cd2—O11—Na2	165.0 (3)	Cd2—O12—C45—O11	0.9 (6)
O9—Cd2—O11—Na3	9.84 (13)	Cd2—O12—C45—C46	−178.9 (5)
O8—Cd2—O11—Na3	−100.02 (13)	Na2—O11—C45—O12	97.3 (6)
N3—Cd2—O11—Na3	−161.4 (2)	Na3—O11—C45—O12	−98.5 (6)
O12—Cd2—O11—Na3	137.0 (2)	Cd2—O11—C45—O12	−0.8 (5)
N4—Cd2—O11—Na3	71.4 (3)	Na2—O11—C45—C46	−82.8 (6)
O9—Cd2—O12—C45	56.5 (4)	Na3—O11—C45—C46	81.3 (7)
O8—Cd2—O12—C45	−61.3 (4)	Cd2—O11—C45—C46	179.0 (5)
N3—Cd2—O12—C45	−147.0 (3)	C49—O13—C48—C47	76.3 (19)
N4—Cd2—O12—C45	139.9 (4)	Na2—O13—C48—C47	−91.8 (11)
O11—Cd2—O12—C45	−0.5 (3)	C48—O13—C49—O13'	−103 (4)
O11—Na2—O13—C49	125.7 (8)	Na2—O13—C49—O13'	65 (3)
O14—Na2—O13—C49	29.5 (6)	C48—O13—C49—C50	−11.0 (19)
O8—Na2—O13—C49	−153.8 (7)	Na2—O13—C49—C50	157.3 (3)
O18—Na2—O13—C49	32.7 (12)	C48—O13—C49—C54	158.7 (13)
O13'—Na2—O13—C49	−72 (2)	Na2—O13—C49—C54	−32.9 (9)
O7—Na2—O13—C49	−89.1 (8)	O13'—C49—C50—C51	−173.3 (8)
O11—Na2—O13—C48	−63.0 (9)	C54—C49—C50—C51	0.0
O14—Na2—O13—C48	−159.2 (10)	O13—C49—C50—C51	169.4 (8)
O8—Na2—O13—C48	17.5 (9)	C49—C50—C51—C52	0.0
O18—Na2—O13—C48	−156.0 (6)	C50—C51—C52—C53	0.0
O13'—Na2—O13—C48	100 (3)	C51—C52—C53—C54	0.0
O7—Na2—O13—C48	82.1 (9)	C51—C52—C53—C55	−175.5 (5)
O15—Cd3—O14—C54	135.1 (4)	Cd3—O14—C54—C53	7.0 (5)
O17—Cd3—O14—C54	−109.5 (4)	Na2—O14—C54—C53	−159.0 (2)
N6—Cd3—O14—C54	21.5 (6)	Cd3—O14—C54—C49	−174.6 (3)
N5—Cd3—O14—C54	−2.0 (4)	Na2—O14—C54—C49	19.5 (4)
O15—Cd3—O14—Na2	−57.65 (18)	C52—C53—C54—O14	178.4 (4)
O17—Cd3—O14—Na2	57.77 (19)	C55—C53—C54—O14	−6.6 (6)
N6—Cd3—O14—Na2	−171.2 (3)	C52—C53—C54—C49	0.0
N5—Cd3—O14—Na2	165.2 (2)	C55—C53—C54—C49	174.9 (6)
O11—Na2—O14—C54	−116.5 (3)	O13'—C49—C54—O14	−5.0 (8)
O18—Na2—O14—C54	155.2 (3)	C50—C49—C54—O14	−178.6 (4)
O13'—Na2—O14—C54	−18.1 (4)	O13—C49—C54—O14	11.4 (8)
O7—Na2—O14—C54	52.5 (3)	O13'—C49—C54—C53	173.6 (8)
O13—Na2—O14—C54	−26.5 (4)	C50—C49—C54—C53	0.0
O11—Na2—O14—Cd3	74.19 (16)	O13—C49—C54—C53	−170.0 (7)
O18—Na2—O14—Cd3	−14.08 (16)	C56—N5—C55—C53	−178.4 (11)
O13'—Na2—O14—Cd3	172.6 (4)	C56'—N5—C55—C53	−164.9 (11)
O7—Na2—O14—Cd3	−116.81 (16)	Cd3—N5—C55—C53	5.2 (14)
O13—Na2—O14—Cd3	164.2 (4)	C52—C53—C55—N5	175.5 (8)
O14—Cd3—O15—C65	−152.4 (3)	C54—C53—C55—N5	0.3 (12)
O17—Cd3—O15—C65	96.5 (4)	C55—N5—C56—C58	−157.2 (11)
N6—Cd3—O15—C65	0.6 (4)	C56'—N5—C56—C58	147 (6)
N5—Cd3—O15—C65	−51.3 (4)	Cd3—N5—C56—C58	19.7 (15)
O14—Cd3—O15—Na3	52.04 (18)	C55—N5—C56—C57	89.7 (13)
O17—Cd3—O15—Na3	−59.09 (17)	C56'—N5—C56—C57	34 (5)
N6—Cd3—O15—Na3	−154.9 (2)	Cd3—N5—C56—C57	−93.4 (11)
N5—Cd3—O15—Na3	153.2 (2)	C59—N6—C58—C56	−125.8 (11)
O9—Na3—O15—C65	27.2 (8)	C58'—N6—C58—C56	−47 (3)
O11—Na3—O15—C65	132.8 (3)	Cd3—N6—C58—C56	61.3 (10)
O10—Na3—O15—C65	−37.4 (3)	N5—C56—C58—N6	−53.8 (15)
O18—Na3—O15—C65	−140.7 (3)	C57—C56—C58—N6	60.4 (14)
O16—Na3—O15—C65	34.2 (3)	C58'—N6—C59—C60	167.3 (9)
O9—Na3—O15—Cd3	−172.4 (6)	C58—N6—C59—C60	−169.3 (8)
O11—Na3—O15—Cd3	−66.86 (17)	Cd3—N6—C59—C60	1.9 (10)
O10—Na3—O15—Cd3	123.03 (17)	N6—C59—C60—C61	177.6 (6)
O18—Na3—O15—Cd3	19.70 (15)	N6—C59—C60—C65	−8.7 (8)
O16—Na3—O15—Cd3	−165.37 (18)	C65—C60—C61—C62	0.0
O15—Na3—O16—C64	−35.2 (2)	C59—C60—C61—C62	174.0 (4)
O9—Na3—O16—C64	143.3 (3)	C60—C61—C62—C63	0.0
O11—Na3—O16—C64	−135.0 (3)	C61—C62—C63—C64	0.0
O10—Na3—O16—C64	78.8 (3)	C66—O16—C64—C63	9.2 (5)
O18—Na3—O16—C64	−25.6 (4)	Na3—O16—C64—C63	−148.61 (19)
O15—Na3—O16—C66	170.0 (5)	C66—O16—C64—C65	−166.8 (4)
O9—Na3—O16—C66	−11.6 (5)	Na3—O16—C64—C65	35.4 (3)
O11—Na3—O16—C66	70.1 (5)	C62—C63—C64—O16	−175.9 (4)
O10—Na3—O16—C66	−76.1 (5)	C62—C63—C64—C65	0.0
O18—Na3—O16—C66	179.6 (4)	Cd3—O15—C65—C64	177.5 (2)
O15—Cd3—O17—C68	55.4 (4)	Na3—O15—C65—C64	−28.9 (3)
O14—Cd3—O17—C68	−63.4 (4)	Cd3—O15—C65—C60	−6.5 (5)
N6—Cd3—O17—C68	138.5 (4)	Na3—O15—C65—C60	147.1 (2)
N5—Cd3—O17—C68	−147.3 (4)	O16—C64—C65—O15	−7.6 (3)
O11—Na2—O18—C68	−167.6 (5)	C63—C64—C65—O15	176.2 (3)
O14—Na2—O18—C68	−70.6 (5)	O16—C64—C65—C60	176.2 (3)
O8—Na2—O18—C68	112.9 (5)	C63—C64—C65—C60	0.0
O13'—Na2—O18—C68	−58.7 (9)	C61—C60—C65—O15	−175.8 (3)
O7—Na2—O18—C68	45.0 (5)	C59—C60—C65—O15	10.8 (4)
O13—Na2—O18—C68	−73.6 (8)	C61—C60—C65—C64	0.0
O11—Na2—O18—Na3	2.16 (13)	C59—C60—C65—C64	−173.4 (4)
O14—Na2—O18—Na3	99.18 (15)	C64—O16—C66—C67	165.2 (4)
O8—Na2—O18—Na3	−77.36 (15)	Na3—O16—C66—C67	−42.3 (6)
O13'—Na2—O18—Na3	111.1 (7)	Na2—O18—C68—O17	84.6 (7)
O7—Na2—O18—Na3	−145.18 (13)	Na3—O18—C68—O17	−79.3 (9)
O13—Na2—O18—Na3	96.2 (6)	Na2—O18—C68—C69	−95.3 (8)
O15—Na3—O18—C68	60.7 (6)	Na3—O18—C68—C69	100.8 (7)
O9—Na3—O18—C68	−116.5 (6)	Cd3—O17—C68—O18	1.7 (7)
O11—Na3—O18—C68	165.4 (6)	Cd3—O17—C68—C69	−178.3 (6)
O10—Na3—O18—C68	−45.9 (6)	C70'—O19—C70—C71	61 (2)
O16—Na3—O18—C68	51.9 (7)	C50—C49—O13'—C48'	−16.4 (13)
O15—Na3—O18—Na2	−106.85 (15)	C54—C49—O13'—C48'	170.1 (7)
O9—Na3—O18—Na2	75.91 (16)	O13—C49—O13'—C48'	81 (4)
O11—Na3—O18—Na2	−2.16 (13)	C50—C49—O13'—Na2	162.5 (4)
O10—Na3—O18—Na2	146.55 (16)	C54—C49—O13'—Na2	−11.0 (10)
O16—Na3—O18—Na2	−115.7 (3)	O13—C49—O13'—Na2	−100 (4)
O2—Cd1—N1—C9	11.6 (7)	O11—Na2—O13'—C49	107.6 (8)
O3—Cd1—N1—C9	119.1 (7)	O14—Na2—O13'—C49	14.5 (7)
N2—Cd1—N1—C9	171.3 (8)	O8—Na2—O13'—C49	−169.9 (8)
O6—Cd1—N1—C9	−94.6 (7)	O18—Na2—O13'—C49	1.6 (14)
O5—Cd1—N1—C9	−46.6 (9)	O7—Na2—O13'—C49	−107.6 (9)
O2—Cd1—N1—C10'	−151.9 (9)	O13—Na2—O13'—C49	90 (3)
O3—Cd1—N1—C10'	−44.4 (9)	O11—Na2—O13'—C48'	−73.9 (14)
N2—Cd1—N1—C10'	7.9 (9)	O14—Na2—O13'—C48'	−167.0 (15)
O6—Cd1—N1—C10'	101.9 (9)	O8—Na2—O13'—C48'	8.6 (14)
O5—Cd1—N1—C10'	149.9 (8)	O18—Na2—O13'—C48'	−179.9 (9)
O2—Cd1—N1—C10	−170.5 (8)	O7—Na2—O13'—C48'	70.8 (13)
O3—Cd1—N1—C10	−63.1 (8)	O13—Na2—O13'—C48'	−92 (3)
N2—Cd1—N1—C10	−10.8 (8)	C9—N1—C10'—C11'	162.3 (10)
O6—Cd1—N1—C10	83.3 (8)	C10—N1—C10'—C11'	41 (3)
O5—Cd1—N1—C10	131.2 (7)	Cd1—N1—C10'—C11'	−31.4 (15)
O2—Cd1—N2—C13	−115.5 (8)	C13—N2—C11'—C10'	129.8 (11)
O3—Cd1—N2—C13	−6.3 (8)	C12—N2—C11'—C10'	−123 (3)
N1—Cd1—N2—C13	−152.9 (9)	Cd1—N2—C11'—C10'	−42.7 (12)
O6—Cd1—N2—C13	105.4 (9)	C13—N2—C11'—C12'	−120.0 (11)
O5—Cd1—N2—C13	58.5 (10)	C12—N2—C11'—C12'	−12 (3)
O2—Cd1—N2—C12	78.8 (8)	Cd1—N2—C11'—C12'	67.4 (11)
O3—Cd1—N2—C12	−172.1 (7)	N1—C10'—C11'—N2	48.1 (15)
N1—Cd1—N2—C12	41.3 (7)	N1—C10'—C11'—C12'	−63.9 (15)
O6—Cd1—N2—C12	−60.4 (7)	C32—N3—C33'—C34'	133.5 (9)
O5—Cd1—N2—C12	−107.2 (7)	C33—N3—C33'—C34'	56.4 (13)
O2—Cd1—N2—C11'	55.6 (9)	Cd2—N3—C33'—C34'	−63.9 (8)
O3—Cd1—N2—C11'	164.7 (8)	C36—N4—C34'—C33'	171.8 (8)
N1—Cd1—N2—C11'	18.1 (8)	C35—N4—C34'—C33'	46.1 (12)
O6—Cd1—N2—C11'	−83.6 (8)	Cd2—N4—C34'—C33'	−31.9 (11)
O5—Cd1—N2—C11'	−130.4 (7)	C36—N4—C34'—C35'	−71.6 (13)
O9—Cd2—N3—C32	−116.0 (6)	C35—N4—C34'—C35'	163 (2)
O8—Cd2—N3—C32	−9.7 (6)	Cd2—N4—C34'—C35'	84.7 (12)
O12—Cd2—N3—C32	98.4 (7)	N3—C33'—C34'—N4	62.0 (11)
N4—Cd2—N3—C32	−164.5 (7)	N3—C33'—C34'—C35'	−68.1 (11)
O11—Cd2—N3—C32	50.9 (8)	C55—N5—C56'—C58'	−166.9 (10)
O9—Cd2—N3—C33	54.8 (8)	C56—N5—C56'—C58'	−37 (5)
O8—Cd2—N3—C33	161.1 (7)	Cd3—N5—C56'—C58'	22.0 (16)
O12—Cd2—N3—C33	−90.8 (7)	C55—N5—C56'—C57'	77.9 (16)
N4—Cd2—N3—C33	6.2 (7)	C56—N5—C56'—C57'	−152 (6)
O11—Cd2—N3—C33	−138.4 (7)	Cd3—N5—C56'—C57'	−93.2 (15)
O9—Cd2—N3—C33'	82.3 (6)	C41—O10—C43'—C44'	165.7 (11)
O8—Cd2—N3—C33'	−171.4 (5)	C43—O10—C43'—C44'	−13 (3)
O12—Cd2—N3—C33'	−63.3 (5)	Na3—O10—C43'—C44'	−50.5 (17)
N4—Cd2—N3—C33'	33.8 (5)	C49—O13'—C48'—C47'	−154.6 (10)
O11—Cd2—N3—C33'	−110.8 (5)	Na2—O13'—C48'—C47'	26.8 (17)
O9—Cd2—N4—C36	0.8 (7)	C59—N6—C58'—C56'	−128.0 (13)
O8—Cd2—N4—C36	105.6 (7)	C58—N6—C58'—C56'	119 (4)
N3—Cd2—N4—C36	150.8 (8)	Cd3—N6—C58'—C56'	39.7 (15)
O12—Cd2—N4—C36	−109.4 (7)	N5—C56'—C58'—N6	−39.9 (17)
O11—Cd2—N4—C36	−60.2 (8)	C57'—C56'—C58'—N6	86.6 (17)
O9—Cd2—N4—C34'	−150.9 (8)	C70—O19—C70'—C71'	−108 (3)

Symmetry code: (i) −*x*+2, −*y*+1, −*z*+1.

### Hydrogen-bond geometry (Å, º)

**Table:**

*D*—H···*A*	*D*—H	H···*A*	*D*···*A*	*D*—H···*A*
O19—H19···O17	0.84	1.99	2.730 (7)	146

## References

[bb1] Barbour, L. J. (2001). *J. Supramol. Chem.* **1**, 189–191.

[bb2] Bruker (2009). *APEX2* and *SAINT* Bruker AXS Inc., Madison, Wisconsin, USA.

[bb3] Fun, H.-K., Kia, R., Kargar, H. & Jamshidvand, A. (2009). *Acta Cryst.* E**65**, o722–o723. 10.1107/S1600536809008137 PMC2968989 21582458

[bb4] Jia, Z. (2009). *Acta Cryst.* E**65**, o646. 10.1107/S1600536809003328 PMC2968515 21582294

[bb5] Sheldrick, G. M. (1996). *SADABS* University of Göttingen, Germany.

[bb6] Sheldrick, G. M. (2008). *Acta Cryst.* A**64**, 112–122. 10.1107/S0108767307043930 18156677

[bb7] Westrip, S. P. (2010). *J. Appl. Cryst.* **43**, 920–925.

